# Recent progress in ROS-responsive biomaterials for the diagnosis and treatment of cardiovascular diseases

**DOI:** 10.7150/thno.106991

**Published:** 2025-04-11

**Authors:** Zhiyu Yuan, Ying Li, Ming Sun, Mujie Yuan, Zeyu Han, Xiaojing Li, Song Liu, Yong Sun, Jie Cao, Fan Li

**Affiliations:** 1 Department of Oral Implantology, The Affiliated Hospital of Qingdao University, Qingdao University, Qingdao 266000, China.; 2 Department of Cardiology, The Affiliated Hospital of Qingdao University, Qingdao University, Qingdao 266000, China.; 3 Department of Pharmaceutics, Qingdao University School of Pharmacy, Qingdao 266021, China.

**Keywords:** Cardiovascular diseases, Theranostics, ROS-responsive, Diagnosis

## Introduction

Cardiovascular diseases (CVDs), driven by factors such as atherosclerosis (AS), hypertension, and hyperlipidemia, pose a significant global health burden 1. These diseases can lead to ischemic or hemorrhagic lesions in the heart and other tissues, resulting in significant morbidity, disability, and mortality. The rising incidence of CVDs, driven by changing lifestyles and an aging population, constitutes a major public health threat 2,3. Early and accurate diagnosis is paramount in CVDs management. For example, unstable plaques, characterized by thin fibrous caps and large lipid cores, are a hallmark of AS. These vulnerable plaques are prone to rupture, leading to thrombus formation and subsequent acute coronary syndromes or stroke 4. Advanced diagnostic tools that enable early detection of these vulnerable plaques can significantly reduce the risk of AS-related complications and facilitate personalized treatment approaches 5. Accurate diagnosis not only targets the root cause of CVDs more effectively but also minimizes unnecessary side effects, ultimately leading to improved treatment outcomes. Currently, clinical management of CVDs primarily involves medication, interventional procedures, and surgical interventions. Pharmacological treatment typically utilizes beta-blockers, statins, and antiplatelet agents, which have proven effective in controlling symptoms and slowing disease progression 6. However, their short half-lives and low bioavailability limit their long-term efficacy. When medication fails to achieve desired results or patient needs are unmet, interventional procedures, such as stent implantation and percutaneous coronary intervention (PCI), become the preferred approach. These methods offer rapid symptom relief, blood flow restoration, and minimal invasiveness. However, for patients with complex conditions or limited response to medication and interventional therapies, surgical intervention provides a more direct treatment option, enabling repair or replacement of damaged cardiac tissues or vessels. While these treatment modalities offer distinct advantages, they also have limitations. Treatment efficacy varies among individuals, and some surgical procedures are associated with significant trauma and prolonged recovery times 7. Therefore, developing safer and more effective treatment methods for CVDs remains a critical challenge.

The advent of smart, responsive biomaterials has ushered in a new era of personalized medicine for CVDs management 8,9. Among these innovations, reactive oxygen species (ROS)-responsive biomaterials hold significant promise as targeted, condition-responsive treatments for CVDs 10,11. ROS, including superoxide anion, hydrogen peroxide (H_2_O_2_), and hydroxyl radical (·OH), play a critical role in cellular signaling and physiological regulation under normal physiological conditions 12. A delicate balance between ROS generation and scavenging exists within cells, ensuring their appropriate involvement in cellular processes. However, under pathological conditions, ROS production significantly escalates, exceeding the cell's capacity for detoxification and leading to oxidative stress. This excess ROS can trigger cell apoptosis, inflammation, and tissue damage. Within the pathological microenvironment of CVDs, elevated ROS levels contribute to oxidative stress, endothelial dysfunction, inflammation, and AS 13. ROS-responsive biomaterials contain ROS-responsive groups that react to ROS, leading to changes in solubility or degradation at the structural level. This process can lower ROS levels, release encapsulated diagnostic or therapeutic agents, and alleviate pathological processes, thereby offering unique advantages in the treatment of CVDs 14. Furthermore, ROS-responsive biomaterials offer several key advantages for CVDs treatment: 1. Precision-Controlled Release: ROS-responsive biomaterials enable controlled drug or probe release in oxidative stress environments, enhancing therapeutic efficacy and minimizing systemic side effects. 2. Oxidative Stress Alleviation: ROS-responsive biomaterials regulate the release of therapeutic and diagnostic agents while simultaneously reacting with ROS, mitigating pathological damage and contributing to CVD management. 3. Combined Diagnosis and Therapy: ROS-responsive biomaterials can be engineered to encapsulate fluorescent agents or nanoparticles, enabling dual functionalities for both diagnosis and treatment, offering a comprehensive approach to CVDs management.

**Figure 1:**
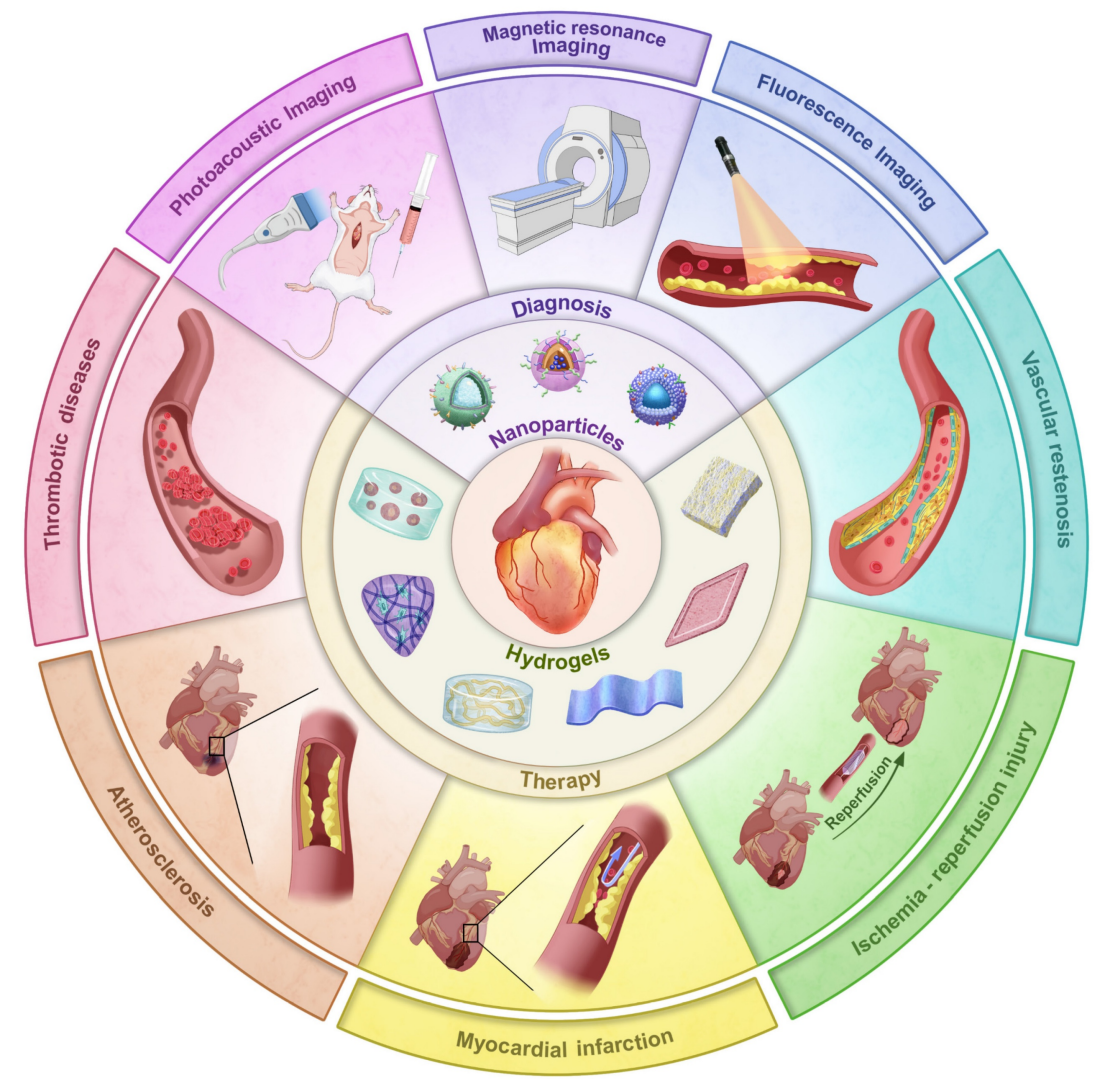
Illustration of ROS-responsive nanoparticles and hydrogels in the diagnosis and treatment of different CVDs.

Despite extensive research exploring the application of ROS-responsive biomaterials in CVDs, a comprehensive review remains lacking. This review fills this gap, providing a comprehensive overview of the application of ROS-responsive biomaterials in the early diagnosis and treatment of CVDs **(Figure 1)**. Firstly, we analyze the key responsive groups and mechanisms of ROS-responsive materials, such as ROS-induced solubility changes and material degradation, and present common ROS-responsive biomaterials used in CVDs. We then delve into the multifaceted role of ROS in various CVDs, including thrombosis, AS, myocardial infarction (MI), ischemia-reperfusion injury (I/R injury), and restenosis, focusing on recent advancements in the diagnosis and treatment of these diseases using ROS-responsive biomaterials. Finally, we discuss the future prospects of this field, providing new directions and insights for future research. To provide a clear foundation for understanding the detailed mechanisms, the underlying biochemical and biophysical interactions that govern ROS responsiveness in biomaterials are first explored. This will set the stage for discussing how these materials can be leveraged to address specific challenges in the diagnosis and treatment of CVDs.

## ROS-responsive biomaterials and their response mechanism

CVDs are characterized by increased oxidative stress, with excessive ROS playing a crucial role in their pathogenesis and progression. This makes ROS-responsive biomaterials a promising theranostic approach for CVDs. Understanding the mechanisms of ROS response and the types of materials that can respond to ROS are critical for diagnosing and treating CVDs. Currently, materials that used for ROS responsiveness mainly include nanoparticles and hydrogels. These materials achieve responsive degradation and drug release through ROS-responsive bonds or groups that enable hydrophobic-to-hydrophilic transitions and redox-triggered degradation, thereby facilitating both the diagnosis and treatment of CVDs.

### Response mechanism of ROS-responsive biomaterials

**Figure 2:**
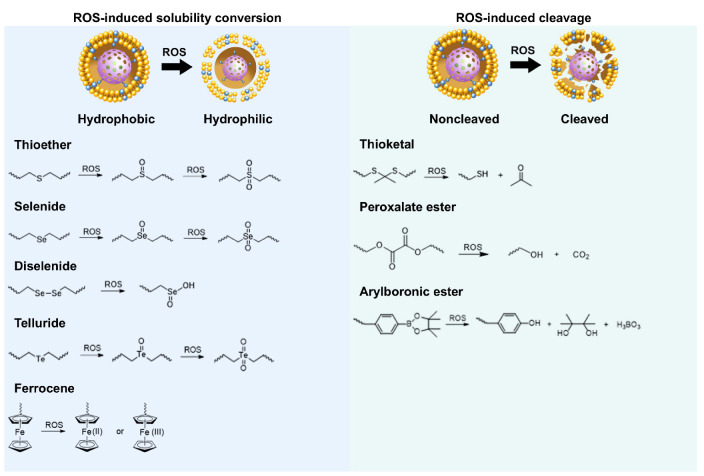
Response mechanism of ROS-responsive biomaterials.

The core of ROS-responsive biomaterials is the presence of ROS-responsive groups (also known as ROS-responsive linkers) incorporated into their structures 15. These ROS-responsive biomaterials can react specifically to ROS after being modified with ROS-responsive groups, which leads to the breakage or polarity change of their chain segments, thereby regulating the drug release from the carriers. ROS-responsive groups can be divided into two categories based on their reaction mechanism: those that respond to ROS by inducing solubility conversion of biomaterials, and those that respond to ROS by inducing degradation of biomaterials 14. The types of groups involved in these two different approaches are detailed below **(Figure 2)**.

### Inducing solubility changes in biomaterials

ROS-responsive biomaterials of this type can transition from a hydrophobic to a hydrophilic state upon activation by high levels of ROS in CVDs. Chalcogen elements (sulfur, selenium, and tellurium) are oxidized by ROS from lower valence states to higher valence states. During this oxidation process, oxygen atoms and chalcogen elements form polar groups that interact with water molecules in the environment, creating hydrogen bonds. Ferrocene (Fc) groups, upon ROS-induced oxidation, are converted into hydrophilic cations. This hydrophobic-to-hydrophilic transition enhances the solubility and stability of drugs, improving drug delivery, particularly to targets such as atherosclerotic plaques or ischemic tissues. However, challenges remain in controlling drug release and ensuring the stability of biomaterials *in vivo*, which may limit the effectiveness of long-term treatments.

#### Thioether

Hydrophobic biomaterials containing thioether groups can be oxidized to hydrophilic sulfoxide or sulfone species in the presence of H_2_O_2_. Specifically, thioether groups form sulfoxide under mild oxidation and sulfone under stronger conditions, enabling targeted ROS-response 16. The main types of thioether-containing biomaterials include polypropylene sulfide (PPS) and phenyl sulfide, among others. In 2004, Hubbell and colleagues reported an amphiphilic triblock copolymer synthesized using a hydrophobic polypropylene thioether segment and a hydrophilic polyethylene glycol (PEG) segment, which was also the first report of ROS-responsive biomaterials 17. This copolymer can self-assemble in water to form ROS-responsive, U-shaped vesicles for drug delivery applications. The copolymer was efficiently degraded in the presence of 10% H_2_O_2_. Yu et al. synthesized a novel ROS-responsive carrier by using carboxymethyl chitosan and methionine 18. The natural organic compound methionine contained a ROS-responsive thioether bond. Under the action of ROS, the thioether was oxidized to a hydrophilic sulfone, resulting in the release of the encapsulated astaxanthin drug. This design demonstrates the potential of exploiting the hydrophobic-to-hydrophilic phase transition mechanism of thioether-containing biomaterials for drug delivery applications.

#### Selenide

Selenides are similar to thioethers and can undergo a transition from a hydrophobic state to a hydrophilic sulfone or sulfoxide species in response to ROS 19. Selenium possesses high reactivity and sensitivity due to its low electronegativity and large atomic radius. He et al. investigated a RAP-loaded polydiselenide micelle hydrogel (PSeR) that self-assembled into SeSe nano-micelles in phosphate buffered saline and exhibited high sensitivity to ROS, enabling the release of RAP at low H_2_O_2_ concentrations (10 μM) 20. The rapid responsiveness and chemical stability of selenides conferred great advantages for drug delivery applications, as they could precisely respond to ROS in the pathological environment and improve the therapeutic effect. This approach offers new prospects for the treatment of ROS-related diseases, such as CVDs.

#### Telluride

Tellurides possess a lower oxidation potential compared to sulfur and selenium, and exhibit excellent oxidation sensitivity, enabling it to respond to lower concentrations of ROS 21. Li et al. developed nanoparticles based on a novel amphiphilic telluride-containing polymer (PEG-PUTE-PEG), which was coated with both cisplatin and indocyanine green (ICG) 22. Under NIR laser stimulation, the telluride atoms in the nanocarrier were easily oxidized by the ^1^O_2_ generated by ICG, thereby enabling selective drug release in the target tissues. Additionally, ROS-responsive micelles prepared using a hyperbranched telluride-containing polymer (HBPTe1900) exhibited remarkable properties in the ROS response assay 23. In the micelle solution containing 100 μmol/L H_2_O_2_, the particle size increased to 5 times the original size after 24 hours, indicating that the tellurium atoms transitioned from a hydrophobic to a hydrophilic state. In contrast, the particle size of the micelle solution without H_2_O_2_ remained unchanged. These results suggest that HBPTe1900 is highly responsive to low concentrations of H_2_O_2_, demonstrating its potential as a ROS-responsive carrier material.

#### Diselenide

Biomaterials containing diselenides can serve as ROS-responsive drug carriers. When exposed to high ROS levels, diselenide bonds are cleaved, generating selenic acid or selenite. This process degrades the biomaterial and triggers the release of encapsulated drugs 24. These diselenide-containing micelles have been reported to react with ROS, even at low concentrations of 0.01% v/v H_2_O_2_, and rapidly release the drug cargo. For example, Kong et al. designed a multifunctional nanoparticle with a Cu_4.6_O core, coated with zein conjugated to docosahexaenoic acid (DHA) via a diselenide bond, and further coated with a platelet cell membrane (PLM) 25. This design allowed the disruption of the diselenide bond in the ROS-rich environment of ischemic stroke (IS), leading to the destruction of the nanoparticles and subsequent release of Cu_4.6_O and DHA. Weng et al. incorporated the ROS-responsive DSPE-SeSe-PEG2000 into liposomes for the treatment of myocardial ischemia-reperfusion injury (MIRI) 26. Upon exposure to 100 μM H_2_O_2_, this ROS-responsive liposome was able to achieve a localized drug release strategy.

#### Ferrocene

Fc is an organometallic compound with aromatic characteristics, consisting of two symmetrical cyclopentadienes linked on either side of a divalent iron atom. Fc reacts with H_2_O_2_ via the Fenton reaction, leading to the transformation from a hydrophobic to a hydrophilic state. This is due to the fact that Fc can change from its uncharged form to its charged form upon oxidation which has therapeutic applications in the treatment of CVDs 27,28. He et al. grafted Fc onto docosahexaenoic acid (HA) to form HA-Fc nanoparticles, and then cross-linked NP^3^_ST_ via a multivalent host-guest interaction between β-cyclodextrin (β-CD) and Fc to form a ROS-responsive nanoassembly (HA-Fc/NP^3^_ST_) 29. In the intima of plaque lesions, the hydrophobic Fc was oxidized to Fc^+^ under the action of excessive ROS, which leaded to the dissociation of the β-CD/Fc complex and the disintegration of the HA-Fc/NP^3^_ST_ nanoassembly, thereby releasing the NP^3^_ST_ and playing a therapeutic role in AS. Meanwhile, this property of Fc is also widely used for ferroptosis in cancer therapy. For example, Jia et al. designed a cascade bioreactor based on a host-guest molecular inclusion complex (PCFP@PL/p53), which included a hydrophilic polyethyleneimine-ferrocene and a hydrophobic hemirotaxane linked via a ROS-responsive molecular switch β-CD@Fc 30. In the tumor's high ROS environment, this switch was activated, leading to the rapid breakdown of the PCFP@PL/p53 complex.

### Inducing the degradation of biomaterials

In this type of ROS-responsive biomaterials, structures such as thioketal, peroxalate ester, and arylboronic ester undergo bond cleavage upon reaction with ROS, enabling the efficient release of encapsulated imaging agents or drugs in the high ROS environment of CVDs. ROS-responsive degradation provides a strategy for targeting damaged tissues. However, to be effective, the degradation rate must be carefully controlled to prevent premature release or excessive decomposition before reaching the target. Additionally, the potential side effects of the degradation products formed after ROS-triggered bond cleavage must also be thoroughly evaluated to ensure their safety for the human body.

#### Thioketal

Thioketal materials possess substantial reducing capabilities and are readily susceptible to oxidation in environments with high levels of ROS 31. Yao et al. developed a ROS-responsive, biodegradable and elastic PUTK heart patch, in which thioketal bonds are efficiently cleaved by H_2_O_2_, enabling controlled drug release and protecting the myocardium from oxidative damage 32. Polymers containing polythioketal can be synthesized through the direct condensation of thiols and have been employed in the treatment of inflammatory diseases. Exposure of polythioketal nanoparticles to high concentrations of ROS, such as 100 mM H_2_O_2_, resulted in a transition of the polymer backbone from a hydrophobic to a hydrophilic state. Under weakly acidic conditions, such as a pH of 5, the ketal groups within the polymer chains underwent rapid degradation. These nanoparticles can only be fully degraded when exposed to both ROS and low pH conditions, which maximize the release of the encapsulated drugs or proteins. For instance, Xue et al. developed a high-drug loading nanoparticle (BTZ@PTK) that self-assembled from the ROS-responsive poly(thioketal) PTK and the drug bortezomib (BTZ), targeting the treatment of osteoarthritis 33. In the presence of H_2_O_2_, the thioketal bond was degraded, resulting in the release of BTZ and demonstrating favorable drug release characteristics.

#### Peroxalate ester

Peroxalate esters are compounds that can be oxidized by H_2_O_2_, and this oxidation process leads to their breakdown into alcohols and carbon dioxide (CO_2_), which can initiate the breaking of chemical bonds to release drugs or other therapeutic agents 34. For example, Liang et al. designed a novel responsive carrier 6s-PLGA-DAr-PO-PEG, with an outer layer coated with biomimetic nanoparticles prepared from erythrocyte membranes (RPP-PU) 35. In the high ROS microenvironment of AS, the peroxyoxalate bond was oxidatively broken, releasing the loaded drug. Additionally, the conjugation of peroxyoxalate with the benzene ring significantly improved the material's sensitivity to H_2_O_2_ and effectively removed excess H_2_O_2_.

Oxalate compounds can be used not only as drug carriers but also as fluorescent dyes. When the oxalate compounds are mixed with H_2_O_2_, part of the oxalate is converted to 1,2-dioxetanedione, which is unstable and immediately decomposes to produce CO_2_ and release photons, resulting in chemiluminescence 36. Using this principle, CRANAD-61, a near-infrared molecular probe based on a curcumin oxalate derivative was designed and synthesized to detect the concentration of ROS in the brain, from the macroscopic to the microscopic level, particularly around amyloid-beta (Aβ) plaques 37. In addition, Lee et al. proposed a novel theranostic agent: hydroxybenzyl alcohol (HBA)-incorporating polyoxalate copolymer (HPOX) nanoparticles loaded with rubrene (Rb) as a fluorophore (HPOX/Rb), for treating I/R injury 38. Upon the addition of H_2_O_2_ to the HPOX/Rb suspension, the nanoparticles rapidly initiated a chemiluminescence reaction, producing the high-energy intermediate dioxetanedione. This process chemically excised Rb, resulting in a strong light emission at 565 nm, which enabled the detection and quantification of H_2_O_2_ concentrations as low as 250 nM. This study demonstrates the potential of multifunctional nanoparticles in treating ischemia-reperfusion-related diseases, such as cardiovascular and neurovascular conditions.

#### Arylboronic ester

Arylboronic esters can act as ROS-responsive linkers, as they can be oxidized to hydroquinone, pinacol and boric acid. Notably, arylboronic esters are selectively sensitive to H_2_O_2_ and are not oxidized by other types of ROS 39. The reaction kinetics of the arylboronic esters are determined by the nucleophilicity of the boron center. When H_2_O_2_ attacks the phenylboronic ester in a nucleophilic manner, the phenylboronic ester undergoes a Baeyer-Villiger oxidation-like rearrangement. This is followed by hydrolysis, which forms a phenolic hydroxyl group and releases the coated active molecule under physiological conditions 40. The degradation products can be easily excreted from the body through the kidneys. Chen et al. developed the shear-thinning laponite hydrogels containing ROS-responsive SAB-loaded nanoparticles (SAB-NPs) as a drug-delivery vehicle for the treatment of peripheral artery disease (PAD) 41. The boronic ester on SAB-NPs was sensitive to H_2_O_2_, resulting in the complete hydrolysis of 100 μM of the SAB-NPs after 9 minutes in PBS containing 600 μM H_2_O_2_. The hydrolysis of SAB-NPs converted it to a hydrophilic glucan, which leaded to the simultaneous release of the drug. In another study, a series of multi-stage ROS-responsive hydrogels (PPBA-TA-PVA) coupled with natural polyphenols were fabricated using ROS-responsive borate bonds 42. The ROS-responsive borate bonds were used as the crosslinking chemical bonds and anti-inflammatory drug coupling points, which gave the PPBA-TA-PVA hydrogel ROS-responsive degradation and anti-inflammatory drug release properties. The hydrogel can adaptively control the degradation rate and the release rate of anti-inflammatory drugs based on changes in the ROS concentration of the wound, which allows it to meet the therapeutic needs of the wound in real-time.

### ROS-responsive biomaterials

**Figure 3:**
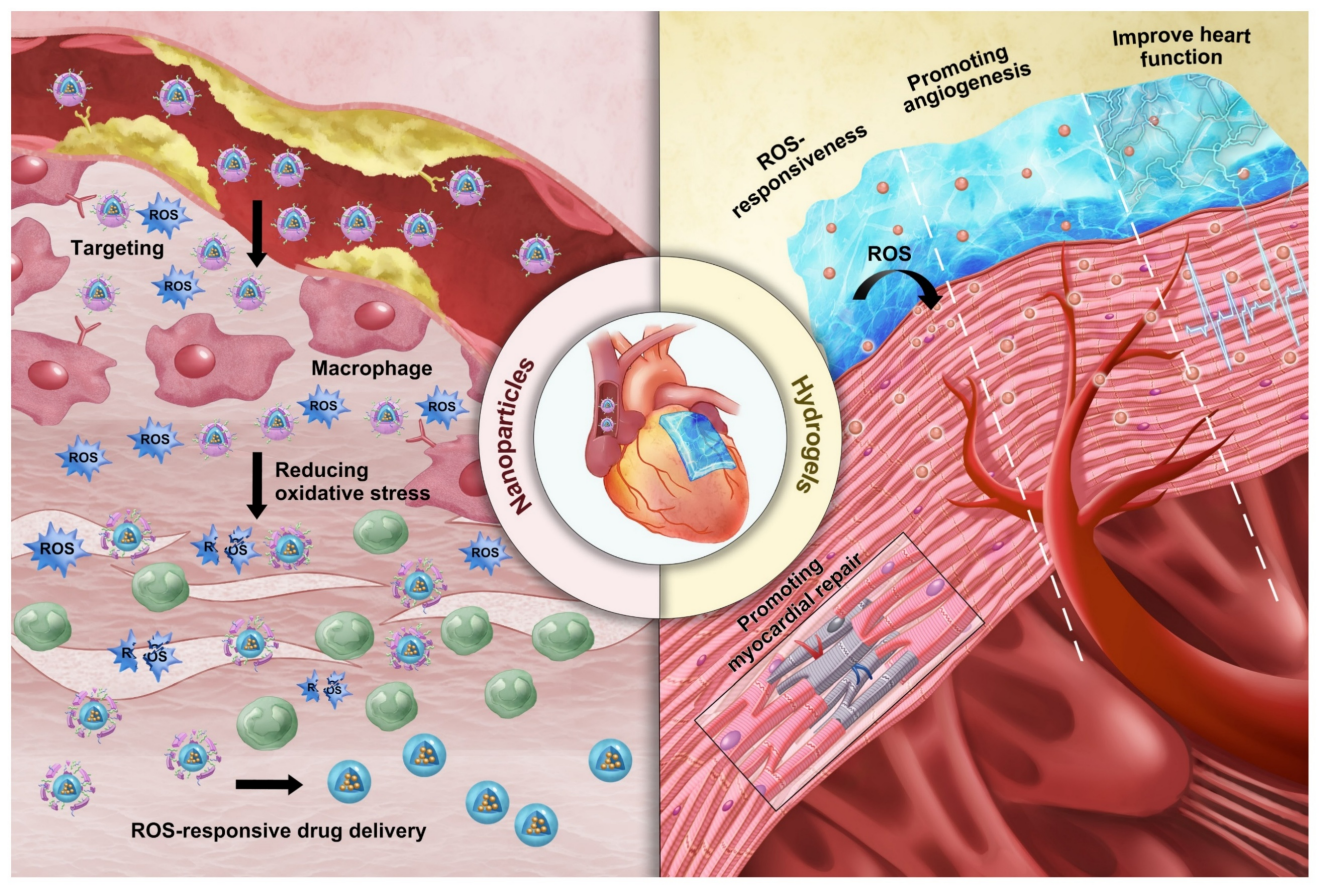
Mechanisms of action of the two ROS-responsive biomaterials.

In recent years, the rapid progress in materials science has enabled ROS-responsive biomaterials to show great potential in the clinical treatment of CVDs. ROS-responsive biomaterials can perform precise drug delivery and intelligent release according to the high ROS levels in the pathological environment, thereby improving the accuracy and efficacy of treatment 43. ROS-responsive biomaterials, such as nanoparticles and hydrogels, are widely used in the fields of drug delivery, cardiac and vascular repair and regeneration, as well as heart valve replacement 44,45,46. Their superior biocompatibility and tunable physicochemical properties provide strong support for the diagnosis and treatment of CVDs **(Figure 3)**. ROS-responsive biomaterials offer several advantages over other internal stimulus-responsive materials in cardiovascular therapy. Unlike traditional drug delivery systems (DDS), they specifically target elevated ROS levels in disease sites, allowing precise drug delivery and reducing side effects. They also respond rapidly to acute events like MI, ensuring timely drug release. In comparison to external stimulus-based systems, ROS-responsive materials can penetrate deep cardiovascular tissues without external interference. Additionally, they not only release therapeutic agents in response to ROS but also reduce excessive ROS, addressing both the symptoms and causes of CVDs. Enzyme- and pH-responsive materials, while useful in certain applications, lack this dual functionality and adaptability to dynamic ROS changes, making ROS-responsive biomaterials more versatile and effective in CVD treatment and diagnosis.

**Figure 4:**
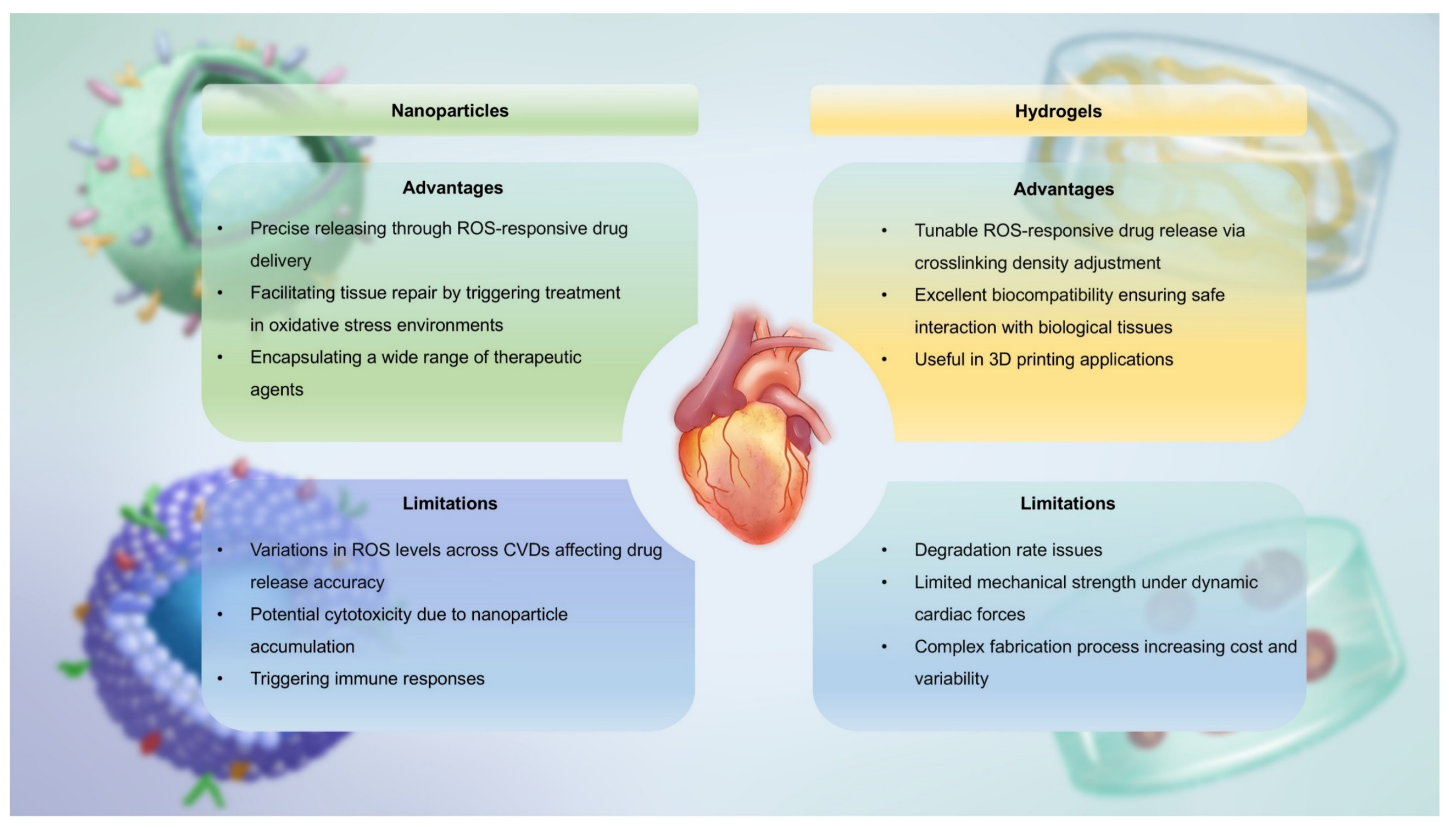
Advantages and limitations of ROS-responsive biomaterials in CVDs.

In this section, we introduce ROS-responsive biomaterials for CVDs diagnosis and treatment, analyzing their advantages and limitations **(Figure 4)**. A deeper understanding of their capabilities is essential for optimizing theranostic strategies and advancing precision medicine in CVDs treatment.

#### Nanoparticles

Nanoparticles are fundamental structures at the nanoscale, typically defined as assemblies of atoms with radii ranging from tens to hundreds of nanometers. Compared to conventional materials, nanoparticles possess unique characteristics such as their nanoscale dimensions, abundant surface functional groups, large surface area, ease of surface modification, and interactions between structural units. These properties enable the tuning of specific functionalities through simple adjustments to the structure, size, shape and composition of the nanoparticles, making them innovative biomaterials for diagnosing and treating CVDs. In recent years, the emergence of “smart” or stimuli-responsive nanoparticles has opened up exciting possibilities for medical treatments 47. Among diverse responsive nanoparticles, ROS-responsive systems have garnered significant attention. This is due to their ability to react to the elevated levels of oxidative stress commonly found in diseased tissues. ROS play a crucial role in numerous pathological conditions, such as inflammation, cancer and CVDs. ROS-responsive nanoparticles are designed to undergo specific structural or behavioral changes in the presence of ROS, which can trigger the release of encapsulated theranostic agents or activate inherent therapeutic properties of the nanoparticle material itself. With the emergence of ROS-responsive nanoparticles, researchers now have more precise and controllable tools, paving the way for advanced theranostic strategies 48.

Nanoparticles have significant advantages in the diagnosis and treatment of CVDs. Firstly, systemic drug delivery often suffers from reduced efficacy due to rapid neutralization. In contrast, nanoparticles, owing to their nanoscale size, can easily traverse biological barriers such as the endothelial layer and directly deliver drugs to damaged sites within the heart and bloodstream 49. This significantly reduces the drug efficacy loss associated with systemic administration, enhancing therapeutic outcomes. Secondly, the high surface-to-volume ratio of nanoparticles allows for efficient encapsulation of cargo and enables precise control over the release of drugs or bioactive molecules at specific locations. Furthermore, modifying the structure of nanoparticles can endow them with superior biomolecular regulatory capabilities 50. For example, coronary artery stent implantation often leads to intimal hyperplasia and in-stent restenosis. Zhang et al. prepared a polymer that releases nitric oxide (NO) by doping gas-phase silica particles into medical-grade polyurethane 51. The NO released by these polymers mimicked the NO flux generated by endothelial cells. This reduced platelet adhesion and activation on the vascular wall. It also minimized the migration and proliferation of vascular smooth muscle cells (VMSC) around the stent. Additionally, the surface of nanoparticles can be functionalized with targeting ligands, imaging probes or cell membrane camouflage to enhance their selectivity for specific tissues and enable real-time monitoring of drug delivery. This multifunctionality enhances the precision of diagnosis and treatment, thereby improving theranostic effects. For instance, Huang et al. encapsulated iron oxide nanoparticles (IONPs) and rapamycin within liposomes, further modifying them with fluorescent agents 52. These nanoparticles, equipped with the targeting peptide VHPKQHR, specifically bounded to VCAM-1 on endothelial cells. They formed a nanoplatform suitable for both magnetic resonance imaging (MRI) and fluorescence bimodal imaging of AS, showing potential for early diagnosis. However, despite their impressive performance in CVDs treatment, nanoparticles also present certain limitations. A major concern is their toxicity 53. While their small size allows efficient tissue penetration, nanoparticles may interact with cellular structures in unexpected ways, potentially disrupting normal cell functions. This raises concerns about their cytotoxicity, particularly with long-term exposure or repeated dosing. Additionally, the* in vivo* stability of nanoparticles remains a critical issue. They are prone to interacting with plasma proteins, which can lead to phagocytosis and rapid clearance by the mononuclear phagocyte system (MPS), reducing their bioavailability, causing premature drug release, and compromising targeting efficacy 54. Another issue is the immunogenicity of nanoparticles, as their presence in the body may trigger immune responses, leading to inflammation or exacerbating existing CVDs conditions 55.

In CVDs treatment, the unique characteristics of ROS-responsive nanoparticles offer new viable options for diagnosis and therapy. First, nanoparticles serve as effective drug delivery carriers that respond to ROS. Systemic administration often leads to reduced drug efficacy due to rapid neutralization; however, nanoparticles can infiltrate cardiac tissue and the bloodstream to directly deliver drugs to the affected areas 49. They respond to elevated ROS levels in the pathological microenvironment and release the drugs accordingly, significantly minimizing the loss of efficacy associated with systemic administration and enhancing treatment outcomes. ROS-responsive polymeric micelle nanoparticles are currently one of the strategies employed for drug delivery in CVDs therapy. These micelles are formed by amphiphilic block polymers in aqueous solutions. They utilize ROS-responsive groups as internal hydrophobic segments to encapsulate hydrophobic drugs, while the external hydrophilic portions enhance overall solubility. Under high ROS concentrations, the responsive groups undergo cleavage, resulting in the release of the encapsulated drugs. Wu et al. developed peptide-amphiphilic nanoassemblies for targeted delivery of responsive drugs 56. The hydrophobic thioether groups in methionine transition to hydrophilic in response to ROS. This structural relaxation controls the release of cargo from the nanoassemblies. This approach enables precise targeting of aging cardiomyocytes and cardiac tissue while minimizing damage to healthy myocardial cells and other organs.

Additionally, by optimizing their surface properties or structures, ROS-responsive nanoparticles can enhance their affinity for CVDs biomarkers, reducing nonspecific adsorption and achieving targeted action in the cardiovascular microenvironment. Moreover, modifying the surface structure of nanoparticles can selectively guide cellular activities, playing a crucial role in CVDs therapy. Ding et al. designed a microreticular nanosystem for myocardial revascularization and repair 57. Hyaluronic acid (HA) provided strong adhesion, allowing the nanosystem to attach to the myocardial surface. CD44, the main receptor for HA, was significantly upregulated after tissue injury, guiding the microreticular nanosystem to target the cardiovascular injury microenvironment. ROS-responsive polycation B-PDEA formed a complex with hypoxia-sensing plasmids (DNA), reacted into elevated ROS levels and underwent hydrolysis, converting to polyacrylic acid and releasing the DNA. After HA was degraded by activated hyaluronidase, it activated macrophages for tissue repair. Furthermore, the multifunctional properties of nanoparticles allow for the creation of nanoplatforms carrying multiple functional groups, leveraging the complementary characteristics of these groups for integrated targeting, imaging, and therapeutic applications. Ni et al. designed a diagnostic and therapeutic nanoplatform that encapsulates anti-Olfr2 siRNA (si-Olfr2) to target macrophages in atherosclerotic lesions and diagnose AS through photoacoustic imaging (PAI) 58. The ROS present in plaque tissue triggered the release of si-Olfr2 from the nanoplatform. Integrating targeting, imaging, and therapy, nanoparticles show immense potential in diagnosing and treating AS while also offering promising applications in personalized medicine and enhancing overall treatment efficacy for various cardiovascular conditions.

#### Hydrogels

Hydrogels are three-dimensional crosslinked networks made primarily of polymer chains and water 59. Since Wichterle and Lim synthesized poly(hydroxyethyl methacrylate) hydrogels in the 1950s, research on the design and synthesis of polymeric hydrogels has rapidly advanced 60,61. Recently, efforts have focused on creating novel hydrogels and exploring their applications in biomedical fields such as drug delivery, tissue scaffolding, and active cell encapsulation 62. In drug delivery, smart responsive hydrogels designed based on the pathological characteristics of targeted lesions have shown significant promise. Compared to conventional drug-delivering hydrogels, these intelligent systems can significantly improve therapeutic outcomes by enabling precise on-demand drug release at disease sites, reducing dosing frequency and side effects. Currently, ROS-responsive hydrogels are widely utilized in disease treatment 63.

Although hydrogels are insoluble in water, they can absorb and retain large amounts of moisture owing to their unique physical or chemical crosslinking structures. The reason is that hydrogels contain a large number of hydrophilic groups on their polymer chains, which bind to water molecules, effectively “locking” them in the network structure and maintaining the stability of the polymer network 59. The water-filled network structure allows the crosslinked polymer chains to maintain shape while possessing certain flexibility and fluidic properties. These properties enable hydrogels to maintain integrity and functionality in solution or within the biological environment. In CVDs, hydrogels also exhibit excellent biocompatibility and drug release capabilities. First, the advantage of hydrogels lies in their ability to simulate the natural environment of human tissues 64. Since most human tissues are composed of protein and polysaccharide networks, which are similar in structure to gel-like hydrated substances, hydrogels can be used as scaffolds in cardiac tissue engineering, providing a microenvironment that supports cell survival, proliferation, and differentiation. Hydrogels can also control the release rate of drugs by adjusting their crosslinking density and hydrophilicity, enabling precise drug-controlled release 65. Moreover, hydrogels are soft and wet materials, making them highly similar to cardiac soft tissue. As a result, they have found wide application in tissue engineering for CVDs. In myocardial tissue repair, hydrogels provide necessary mechanical support and have become essential tools for early clinical treatment of MI 66. For example, clinical trials of Algisyl-LVRTM and IK-5001 hydrogels have demonstrated their ability to provide mechanical support to the left ventricle (LV), inhibiting adverse LV remodeling and significantly improving myocardial function 67. Furthermore, hydrogels can be used in 3D printing technologies to simulate natural myocardial structures, facilitating the repair of myocardial tissue or printing complete myocardial tissue to replace necrotic areas. This approach not only restores myocardial function but also regenerates damaged myocardial tissue to some extent, offering an innovative solution for CVDs treatment 68.

However, hydrogels also face certain challenges. One major issue is their mechanical strength. Although hydrogels can be adjusted for hardness and elasticity to match native tissue, they may still lack sufficient toughness to withstand the dynamic mechanical forces exerted by cardiac contractions and high-pressure blood flow 69. This vulnerability could lead to rupture or deformation of the hydrogel, weakening its long-term effectiveness. Moreover, the degradation rate of hydrogels must be carefully controlled to synchronize with tissue healing processes. Too rapid degradation could lead to premature loss of structural integrity, while too slow degradation could delay the release of therapeutic drugs 70. Achieving this balance is crucial to ensure optimal therapeutic outcomes. Additionally, the manufacturing of hydrogels often involves complex chemical processes, including multiple reaction steps and precise material adjustments. These processes increase production costs and induce variability between batches, undermining the reproducibility and stability of hydrogel-based treatments in clinical applications.

Hydrogels closely resemble soft cardiac tissues in terms of their soft and moist characteristics, making them suitable for various applications in cardiovascular tissue engineering. ROS-responsive hydrogels can serve as carriers for delivering various theranostic agents. Their unique porous structure allows for the embedding of drugs within their pores, either through *in situ* loading or post-loading. Upon exposure to ROS, these hydrogels undergo changes in hydrophobicity and hydrophilicity or polymer chain cleavage, leading to the release of the encapsulated drugs. Moreover, by adjusting their crosslink density and hydrophilicity, the release rate of the drugs can be controlled, facilitating precise drug delivery. In recent years, numerous biodegradable hydrogels capable of loading various functional molecules, including drugs, growth factors, proteins, and genes, have been developed for comprehensive treatment of CVDs. For instance, Hu et al. designed an injectable hydrogel that released curcumin and tailored recombinant humanized collagen type III in a controlled manner under low pH and high ROS conditions, promoting cardiac repair by increasing the expression of cardiac markers such as α-actinin and CX43 71. In addition to serving as drug delivery carriers, ROS-responsive hydrogels incorporating materials with conductive and pro-angiogenic properties can enhance the precision and efficacy of CVDs treatments. For example, conductive hydrogels can improve electrical coupling between cardiomyocytes. Research indicates that integrating conductive materials, such as exosome PPY-CHI/hEMSC-Exo, with hydrogels can restore cardiac electrical transmission, alleviate arrhythmias, and facilitate myocardial repair, significantly enhancing heart function 72. Qiu et al. developed a ROS-responsive injectable conductive hydrogel (BHGD) for treating MI 73. In later stages of MI, the conductive black phosphorus nanosheets (BPNSs) within BHGD facilitated electrophysiological treatment, including compensating for impaired electrical conduction in the infarcted area and restoring cardiac contraction. The ROS-responsive structure protected unstable BPNSs from oxidation, maintaining good conductivity in the MI microenvironment. This hydrogel fosters an environment conducive to intercellular electrical communication and enhances cardiomyocyte maturation. Pro-angiogenic hydrogels also exhibit tremendous potential in treating CVDs. Mechanical plasticity in hydrogels supports vascular endothelial remodeling, which is critical for recovery in CVDs. For example, Wei et al. developed a collagen-hyaluronic acid-based hydrogel platform with tunable mechanical plasticity, promoting vascular endothelial contraction and adhesion, thus enhancing angiogenesis in infarcted areas 74. Zhang et al. created an injectable composite hydrogel scaffold 75. Under high concentrations of ROS, the released Mg^2+^ from hydrogels activated PI3K phosphorylation, stimulating Akt and increasing the expression of vascular endothelial growth factor (VEGF) to promote angiogenesis. As a multifunctional and tunable material, ROS-responsive hydrogels demonstrate vast potential for application in CVDs treatment.

Among the representative examples of functionalized hydrogels, a specific type designed for myocardial repair is known as a cardiac patch 76. These patches facilitate local recovery of damaged or failing myocardial tissue by providing mechanical and regenerative support 77. For patients with CVDs, ROS-responsive heart patches offer a novel and exciting therapeutic option. These patches not only repair damaged cardiac tissue but also promote tissue regeneration through the implantation of embryonic cells or bioactive factors that support heart repair 78. Customizable heart patches tailored to individual patient conditions can address personalized needs, creating multiple possibilities for myocardial tissue repair. One key benefit of ROS-responsive heart patches is their ability to enhance angiogenesis and myocardial cell proliferation, thereby accelerating the repair process of damaged myocardium. For instance, Li and colleagues designed a nanofiber patch with a top layer of hydrophilic PEG molecules to resist fibroblast adhesion, while the bottom layer contained abundant diselenide bonds that mitigated oxidative stress and inflammation at the lesion site in response to ROS 79. Additionally, ROS-responsive heart patches improve myocardial regeneration through the introduction of bioactive factors like active peptides. Yao and colleagues developed a high-strength porous polyurethane heart patch 80. During the early phase of MI, ROS-responsive degradation of PTK released anti-fibrotic rosuvastatin, enhancing the survival of myocardial cells and fostering a pro-angiogenic microenvironment. Combining mechanical support and anti-inflammatory, anti-fibrotic properties in a comprehensive ROS-responsive patch can significantly enhance treatment outcomes for CVDs. Furthermore, advanced technologies such as three-dimensional printing enable the customization of heart patches to meet the specific needs of different patients. This tailored approach allows for better conformability to the heart's surface and shape, improving therapeutic efficacy and patient prognosis 81.

## Diagnosis and therapy of ROS-responsive biomaterials in CVDs

At present, researchers have successfully applied ROS-responsive biomaterials to the diagnosis and treatment of a variety of CVDs, leveraging the design principle of two ROS-responsive groups. In terms of diagnostic imaging, MRI, fluorescence and PAI are the primary focus of current researches on ROS-responsive biomaterials for the diagnosis of CVDs 82,83. In response to excessive ROS in the cardiovascular pathological microenvironment, ROS-responsive biomaterials can achieve the precise release of fluorescent probes or imaging agents, thereby playing a specific diagnostic role. Compared to traditional treatment methods, these ROS-responsive biomaterials emphasize the interaction and dynamic regulation between tissues, and show significant advantages in precision drug delivery, controlled release, mechanical support, and biocompatibility, making them promising candidates for the treatment of CVDs in the future 84,85. In CVDs treatment, mechanisms like antioxidant and anti-inflammatory actions are broadly applicable across various conditions, while some diseases have unique therapeutic mechanisms targeting specific pathological processes. Here, the theranostic approaches for different CVDs are categorized based on their primary mechanisms. Although some treatments may involve multiple mechanisms, we focus on the most prominent one for each disease, with auxiliary mechanisms briefly mentioned to provide a comprehensive overview. This section will first summarize the different application techniques of ROS-responsive biomaterials in the diagnostic imaging of CVDs, and then discuss the role of ROS in various CVDs, as well as the latest research progress of ROS-responsive biomaterials in the treatment mechanisms of different CVDs.

### Diagnosis

Imaging modalities such as MRI, fluorescence, and PAI have been utilized to visualize the structure of the cardiovascular system in patients 82. The increasing popularity of these non-invasive diagnostic techniques has stimulated the continuous development of imaging methods, including advancements in biomaterial-based contrast agents 86. In particular, ROS-responsive biomaterials have demonstrated great potential and advantages in the diagnosis of CVDs. Leveraging the characteristic of abnormally elevated ROS levels in the microenvironment of cardiovascular lesions, ROS-responsive biomaterials can accurately release fluorescent probes or imaging agents in this environment, thereby achieving the function of specific diagnosis.

#### Fluorescence imaging

Fluorescence imaging is a non-invasive method that utilizes fluorescent substances to emit fluorescence under specific wavelength light for real-time, multi-dimensional monitoring of biomolecules, cells, tissues, and organisms 87. This technique offers high sensitivity, high temporal resolution, and non-invasiveness. ROS-responsive biomaterials can be used in the form of loaded imaging agents or small molecule probes to enable the diagnosis of lesion sites. AS is the primary pathological basis of serious CVDs. Rapid identification and detection of vulnerable plaques is the key to timely clinical intervention, reducing mortality and avoiding overtreatment 88. The development of accurate and rapid means or methods for plaque detection is essential for timely and accurate clinical decision-making and early active intervention 89.

A feature of AS is lipid droplets (LDs) accumulation in the intima of arteries. The study on the biological and physiological functions of LDs, such as signal transduction and immune regulation, is significant for understanding AS-related diseases. Liu et al. designed a novel probe named MeOND encapsulated with ROS-responsive nanoscale polymeric micelle (MeOND@PMM). The strong twisted internal charge transfer (TICT) effect of MeOND allowed the probe to show strong fluorescence only in low-polarity solvents while reducing emission in high-polarity aqueous solution. In lipid environment, MeOND was quickly released in the presence of ROS. After treatment with MeOND@PMM, atherosclerotic mice displayed clear plaques in the aortic region, demonstrating that MeOND@PMM exhibited satisfactory LDs-specific imaging in atherosclerotic plaques.

**Figure 5:**
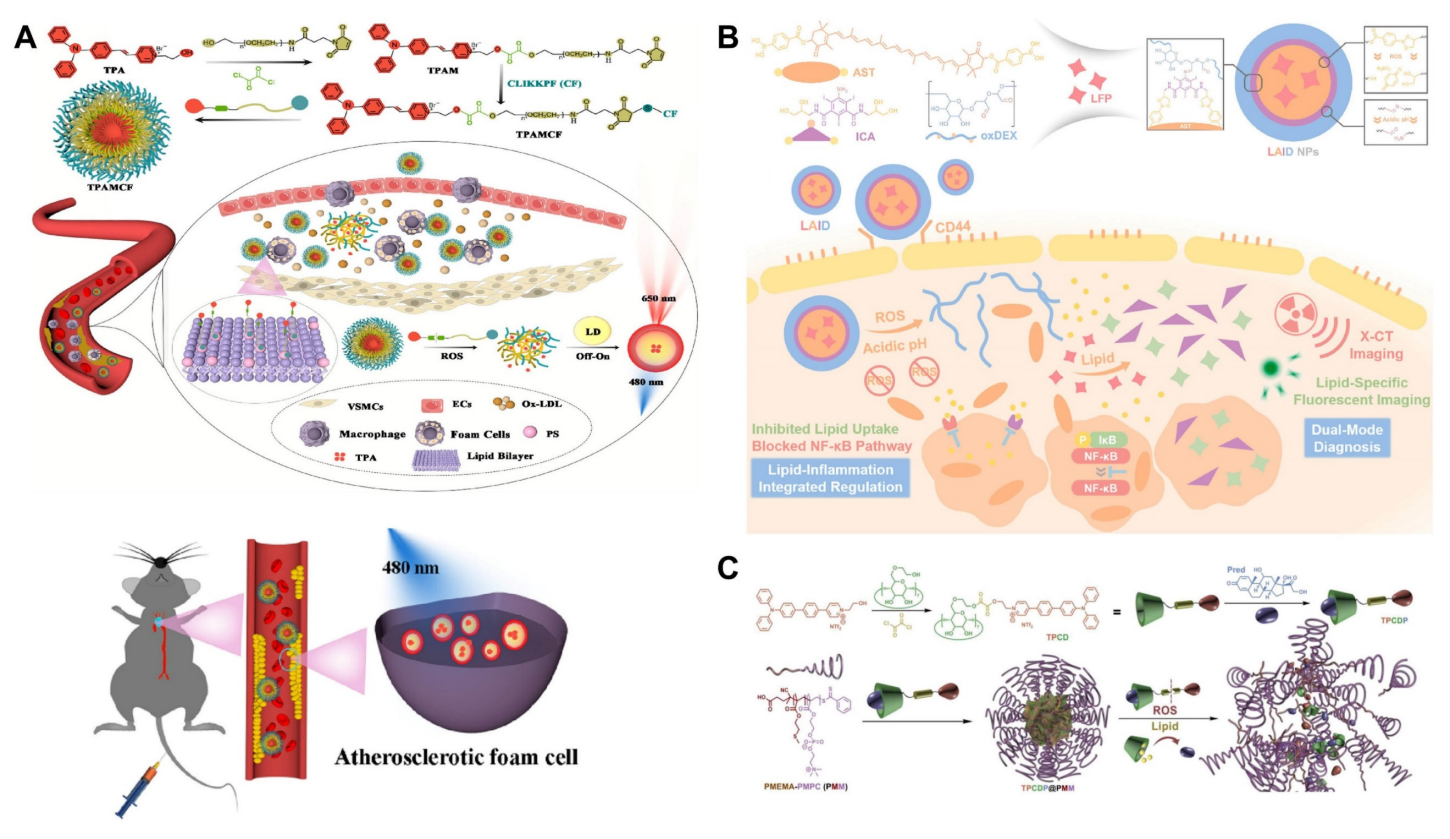
** (A)** Illustration of TPAMCF NPs to Achieve Precise Localization of the Plaques, ROS-Triggered Nanoparticle Disassembly, and AIE Imaging and TPAMCF NPs for imaging of LDs in the aorta. Adapted with permission from 90, copyright 2023, American Chemical Society.** (B)** Illustration of LAID nanoplatform for the theranostics of early-stage vulnerable plaques in AS. Adapted with permission from 92, an open access article published 2020 by John Wiley and Sons under a CC-BY4.0 license. **(C)** Illustration of a theranostic nanoplatform for AS plaque recognition and inhibition. Adapted with permission from 93, copyright 2020, Wiley-VCH GmbH.

Aggregation-induced emission luminophores (AIEgens), a class of fluorescent molecules that do not emit light when dissolved but strongly fluoresce in the aggregated state, have been designed for imaging the lipid site of AS. However, fluorescent probes, such as AIEgens, are generally poorly targeted and can nonspecifically stain other lipid-rich organs and tissues (such as fatty liver and arterial walls). To improve the targeting of AIEgens, Liu et al. developed a ROS-responsive sequentially targeted fluorescent probe (TPAMCF) for AS recognition** (Figure 5A)** 90. The CLIKKPF peptide in the probe could specifically bind to phosphatidylserine from foam cells and accumulate in plaques. Triggered by high concentrations of ROS in local plaque, the oxalate bond in TPAMCF nanoparticles broke, releasing AIEgens that activated and recognized lipid droplets in foam cells, enabling precise localization and fluorescence imaging of AS plaques.

In addition to targeting issues, fluorescent probes are often limited by background interference. To address this, He et al. developed ROS-responsive nanoparticles called HA@PCFT 91. The lipid-specific fluorescent probe (FC-TPA) was encapsulated in the hydrophobic cavity of HA@PCFT. Under high ROS conditions, the hydrophobic FC oxidized and became hydrophilic, causing the material to degrade and release FC-TPA. FC-TPA contained a triphenylamine group, which was lipophilic and effectively bound lipids in plaques, emitting a distinct green fluorescence. To minimize background fluorescence during imaging, the probe fluoresced in non-proton environments, while hydrogen bonds in solvents significantly quenched the fluorescence. This reversible fluorescence switching reduces background interference and improves the signal-to-noise ratio.

Current vascular imaging technologies primarily use a single mode, which cannot fully assess plaque morphology. Therefore, designing dual-mode imaging probes is essential. Wang et al. developed a dual-mode diagnostic and therapeutic nano-platform, LAID, composed of a lipid-specific probe (LFP), boronic acid-modified astaxanthin, iodinated contrast agent, and oxidized dextran (ox-Dex) **(Figure 5B)** 92. In AS plaques, excess ROS and acidic conditions broke the boronic and imine bonds in LAID, causing nanoparticle degradation and releasing ICA, astaxanthin, and LFP encapsulated in the core. In dual-mode imaging experiments, fluorescence imaging showed that LAID emits specific fluorescence at 530 nm, identifying early vulnerable plaques. X-CT imaging revealed strong positive signals from LAID, confirming its ability to localize early plaques. This novel diagnostic agent enables precise identification of early vulnerable plaques, offering new insights for early clinical treatment of AS.

To address the challenges of shallow imaging depth, suboptimal size resolution, and background fluorescence interference in single-photon imaging, Ma et al. developed a theranostic nanoparticle featuring two-photon excitation and aggregation-induced emission (AIE) active fluorophore (TP) **(Figure 5C)** 93. The TP was linked to β-CD via a ROS-responsive bond, while the anti-inflammatory drug prednisone (Pred) entered the CD cavity through supramolecular interactions. This two-photon fluorophore cyclodextrin/prednisone complex (TPCDP) was then encapsulated by an amphiphilic polymer composed of poly (2-methylthioethyl methacrylate) and poly (2-methacryloyloxyethyl phosphorylcholine) (PMEMA-PMPC, PMM) to form micelles (TPCDP@PMM). Under excessive ROS stimulation, the ROS-responsive bond between TP and CD was disrupted, allowing the free TP to facilitate two-photon AIE imaging.* In vivo* two-photon bioimaging studies in AS ApoE^-/-^ mice demonstrated that TPCDP@PMM exhibited significant micelle aggregation and clear resolution for plaque identification, indicating its potential as a promising nanoplatform for integrated diagnosis and therapy.

ROS not only serve as signaling molecules for responsive release of fluorescent agents but are also used in the design of responsive fluorescent probes, which can undergo fluorescence release or enhancement in specific ROS environments. This allows them to detect oxidative stress levels in disease cells or study ROS-related biological processes. Liu et al. developed two RBCM biomimetic ratio-based nanoprobes, which can indicate the presence of AS plaques and accurately reflect the ROS levels at plaque sites 82. The reference component was CH1055, while the small-molecule probes used were HDB and Cy7. The benzeneboronic acid pinacol ester moiety in HDB was oxidized by H_2_O_2_, accompanied by signal enhancement, while the cyanine backbone in Cy7 was oxidized and cleaved by ClO^-^ and ONOO^-^, leading to signal attenuation. The ROS-responsive fluorescent probe not only enabled high-precision indication of plaque presence (fluorescence of CH1055), but also utilized the increase or decrease in fluorescence of HDB or Cy7 to indicate fluctuations in ROS expression. This greatly minimizes interference from nanoprobe accumulation or metabolism on the fluorescence signal, thereby enhancing the accuracy of imaging.

In addition to using lipid droplets in AS as biomarkers for fluorescence imaging, mitochondrial dysfunction is considered one of the main causes of foam cell formation. Accurate and sensitive detection of mitochondrial dysfunction is beneficial for the diagnosis of AS. Wang et al. reported an HCIO-responsive NIR fluorescent probe (AS-CN) for precise detection of AS 94. Under high HCIO levels in foam cells, AS-CN was oxidized to AS-CN-O, which exhibited a blue shift with red emission. Mitochondrial viscosity increased under inflammatory stimulation, and the strong intramolecular charge transfer photophysical process enhanced NIR emission at 710 nm. Thus, the detection of foam cell formation can be achieved from both the physical dimension of viscosity and the chemical dimension of HCIO.

#### Magnetic resonance imaging

MRI is a commonly used clinical imaging technique known for its high spatial resolution, excellent signal-to-noise ratio, and lack of ionizing radiation, making it widely applicable in medical diagnostics. As a non-invasive imaging method for observing and analyzing arterial walls, MRI plays a crucial role in diagnosing CVDs such as AS and vascular inflammation 95. Contrast agents used in MRI enhance image contrast by altering how tissues respond to the magnetic field. These agents improve the visibility of specific tissues or pathological conditions in MRI images, thereby enhancing diagnostic sensitivity and accuracy, particularly in qualitative disease assessment and the detection of small, subtle lesions. T1 and T2 are two distinct imaging weighting methods in MRI, with T1 and T2 contrast agents utilized to enhance the contrast of these weighted images. T1 contrast agents produce a high signal for target tissues in the images, while T2 contrast agents yield a low signal 96. ROS-responsive biomaterials can load T1 or T2 contrast agents to achieve precise release in high ROS areas related to CVDs, further enhancing imaging effectiveness.

Superparamagnetic IONPs are small synthetic particles with a core of Fe_2_O_3_ or Fe_3_O_4_. As a magnetically enhanced T2 contrast agent, they are widely used in biomedical research due to their high sensitivity and ease of surface modification 97. In a study, Wu et al. synthesized a novel core-shell nanoparticle, iron oxide/cerium oxide (IO@CO), by combining cerium dioxide (core) with Fe_2_O_3_ (shell) for the diagnosis and treatment of ROS-related diseases 98. This nanoparticle can be effectively taken up by macrophages in AS. After uptake, cerium dioxide reacted with ROS to lower the ROS levels in macrophages while releasing the iron oxide core, facilitating imaging of the macrophages. Research on *in vitro* MRI of macrophages using IO@CO nanoparticles showed that the relaxation rate of IO@CO-treated macrophages was significantly enhanced compared to untreated macrophages, demonstrating excellent MRI imaging performance.

Carbon fluorescent quantum dots (CDs) are excellent imaging agents due to their high stability and low toxicity 99. When combined with MRI, CDs effectively address the limitations of MRI in distinguishing similar grayscale regions, enabling more accurate and detailed assessment of intravascular plaques. Shen et al. designed a bimodal imaging probe, Fe_3_O_4_@SiO_2_-CDs (FC), which self-assembled with a H_2_O_2_-responsive amphiphilic block copolymer, simvastatin (Sim), modified poly (glycidyl methacrylate)-polyethylene glycol (PGMA-PEG), and the targeting molecule ISO-1, resulting in a drug-loaded micelle, PGMA-PEG-ISO-1-Sim@FC (PPIS@FC) 100. ISO-1 specifically was bound to macrophage migration inhibitory factor, enabling targeted delivery of the drug-loaded micelle. In high ROS environments within atherosclerotic plaques, the oxalyl chloride groups in PGMA-PEG were cleaved, releasing Sim and FC for imaging and treatment of the plaques. *In vivo* magnetic imaging studies showed that as the concentration of FC nanoparticles increased, the MRI signal strengthened, indicating that FC nanoparticles can serve as T2 contrast agents for MRI diagnostics. The design of this bimodal imaging probe helps overcome the limitations of single imaging techniques, offering significant advantages for early diagnosis of AS.

In addition to iron as an MRI contrast agent, manganese can also serve as a T1 contrast agent due to its longer electron relaxation time. Li et al. covalently grafted tempol molecules (capable of scavenging ROS) onto MSN, resulting in TMSN. Subsequently, they coated TMSN with a platelet membrane (PM) to develop a novel nanomedicine, denoted as TMSN@PM 101. When TMSN@PM reached the site of inflammation, it reacted with excess ROS, leading to self-degradation and the release of Mn^2+^. As the concentration of ROS increased, Mn^2+^ exhibited stronger signal intensity in T1-weighted MRI, and this signal intensity showed a nearly linear correlation with H_2_O_2_ concentration. This finding indicates that it is now possible to quantitatively assess ROS levels at inflammation sites using MRI. TMSN@PM can provide real-time feedback on the redox state of affected cardiovascular regions, enabling effective personalized diagnosis and treatment.

Toxicity assessment of metal contrast agents is essential in both their design and clinical application. This includes an evaluation of not only the direct toxic effects but also their metabolism, distribution, excretion, and interactions with other treatments. Ledda et al. studied the metabolism of SPIONs, finding that sub-5 nm SIO-Fl nanoparticles were primarily taken up by the kidneys within two hours of injection 102. They also observed a significant decrease in kidney iron content one week later, suggesting that the kidney was the primary metabolic route for nanoparticles smaller than 5.5 nm in diameter. Wu et al. prepared Fe_3_O_4_ nanoparticles with diameters of 2.3, 4.2, and 9.3 nm, assessing their toxicity in mice after intravenous injection 103. The results showed that at a dose of 100 mg/kg, the ultrasmall Fe_3_O_4_ nanoparticles (2.3 and 4.2 nm) were highly toxic, while the 9.3 nm nanoparticles exhibited no significant toxicity. The 2.3 nm nanoparticles elevated *in vivo* ROS and ·OH levels by inducing ROS and triggering the Fenton reaction, causing oxidative stress in various organs.

The safety of metal contrast agents is particularly critical in the treatment of CVDs, as patients often have conditions such as kidney dysfunction or allergies that may increase toxicity risks. Toxicity evaluation should focus on various aspects, including acute and chronic toxicity, renal toxicity, immunotoxicity, allergic reactions, cellular and molecular toxicity, and biodegradability. This ensures both safety and long-term treatment efficacy. However, limited research exists on the dosage-toxicity relationship of iron and manganese used in MRI, which should be considered in the design of metal-based contrast agents.

#### Photoacoustic Imaging

PAI is an emerging imaging technique 104. When laser light is directed at biological tissues, light-absorbing components within the tissue, such as hemoglobin and melanin, absorb the light energy and convert it into heat, generating acoustic signals. By detecting and processing these acoustic signals, images can be produced that reflect the internal structure and function of the tissue. As a non-invasive imaging technique, PAI offers high sensitivity and deep tissue penetration, making it suitable for the early clinical diagnosis of atherosclerotic plaques 105. Photoacoustic contrast agents enhance imaging contrast and resolution by altering the acoustic and optical properties of the local tissue, thereby assisting PAI 106. ROS-responsive biomaterials can facilitate PAI either by loading photoacoustic contrast agents or by connecting to a scaffold that enables PAI.

**Figure 6:**
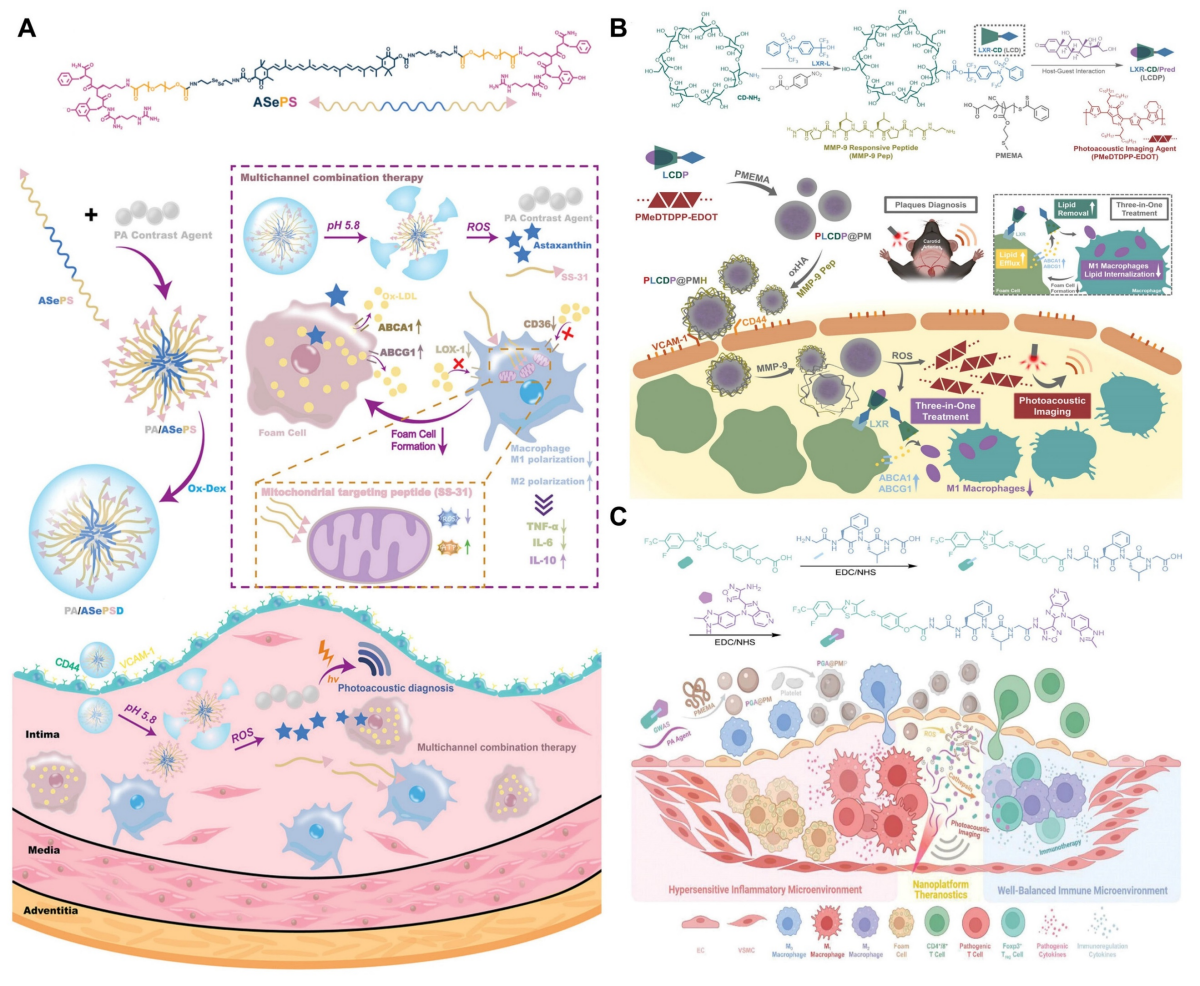
** (A)** Illustration of a dual-response nanoplatform loaded with a photoacoustic contrast agent and equipped with cascade targeting for AS photoacoustic diagnosis and multichannel combination therapy. Adapted with permission from 107, copyright 2023, Wiley-VCH GmbH. **(B)** Illustration of the targeting ROS/MMP responsive PLCDP@PMH theranostic nanoplatform for AS theranostics. Adapted with permission from 108, copyright 2022, Wiley-VCH GmbH. **(C)** Illustration of the PGA@PMP nanoplatform for early-stage AS theranostics, which demonstrates a platelet based active targeting, an overexpressed ROS triggered disintegration, a plaque-specific photoacoustic diagnosis and a CTSB triggered “hand-in-hand” immunoregulation on macrophages polarization and T cells differentiation. Adapted with permission from 109, copyright 2023, Wiley-VCH GmbH.

π-Conjugated polymer probes exhibit excellent PAI properties and have been utilized for deep tissue imaging, presenting a promising option as photoacoustic contrast agent. Xu et al. developed a novel π-conjugated polymer (PMeTPP-MBT) as a photoacoustic contrast agent and constructed a cascade-targeted, dual-responsive nanoplatform, PA/ASePSD, for non-invasive photoacoustic diagnosis and multimodal treatment of AS **(Figure 6A)** 107. Highly hydrophobic astaxanthin molecules were linked to PEG2000 via diselenide bonds, and the mitochondrial-targeting antioxidant peptide SS-31 was introduced, resulting in the formation of the amphiphilic polymer ASePS. Subsequently, a dextran shell was coated onto the nanoplatform through a Schiff base reaction between oxidized dextran (ox-Dex) and the SS-31 peptide, resulting in the preparation of PA/ASePSD. Upon targeting atherosclerotic plaques, the acidic inflammatory microenvironment first triggered the pH-responsive cleavage of the Schiff base, resulting in the shedding of the dextran shell. Concurrently, the diselenide bonds broke under high ROS concentrations, releasing the photoacoustic agent PMeTPP-MBT. This nanoplatform enables non-invasive, real-time diagnosis of early-stage AS. In another study, Ma et al. developed a tri-functional lipid therapeutic complex, LCDP, which was encapsulated with a PAI probe (PMeDTDPP-EDOT) into ROS-responsive nanoparticles (poly (2-methylthioethyl methacrylate), PMEMA) and modified with oxidized hyaluronic acid, resulting in PLCDP@PMH **(Figure 6B)** 108. These nanoparticles respond to both ROS and matrix metalloproteinases (MMP) in AS, enabling dual degradation and the subsequent release of the photoacoustic probe and LCDP complex. The photoacoustic probe demonstrated that specific imaging of lesions in AS mice could accurately and clearly identify plaques. To achieve targeted delivery to AS plaques, Ma et al. developed a nano-platform, PGA@PMP, which loaded the π-conjugated polymer PMeDTDPP-THBTD photoacoustic probe and the immunomodulator GWAS onto the ROS-responsive carrier PMEMA, and was coated with a PM** (Figure 6C)** 109. The integrin proteins on the PM specifically recognized the overexpressed ICAM/VCAM-1 protein on endothelial cells, thereby enhancing the accumulation efficiency of the nanoparticle carrier at the lesion site. *In vivo* PAI of AS was conducted at 6, 12, and 24 hours after the injection of PGA@PMP, revealing clearly visible plaques and a gradual increase in photoacoustic signal over time. Through PM recognition, non-invasive optical diagnostics, and immunomodulation, PGA@PMP enables comprehensive targeted diagnosis and treatment of early-stage AS, providing significant insights for early management of the condition.

PAI activated in the second near-infrared (NIR-II) window (1000-1700 nm) offers significant advantages, including reduced scattering, improved imaging resolution, and increased penetration depth. These characteristics enhance the imaging quality of deep tissues and improve the signal-to-noise ratio, thereby broadening the application of NIR-II PAI in disease diagnosis. Semiconductor polymers with π-conjugated frameworks exhibit great potential in NIR-II PAI due to their high light stability and customizable optical properties. Olfactory receptor 2 (Olfr2) has recently emerged as a potential target for plaque formation. Ni et al. designed si-Olfr2 targeting macrophages and developed a ROS-responsive theranostic platform (si-Olfr2 NPs), which encapsulated si-Olfr2 for targeting macrophages in atherosclerotic lesions, integrating NIR-II PAI functionality with siRNA therapy 58. The semiconductor organic framework SP-PEG formed the outer layer of the nanoparticles, providing stability while supporting PAI functionality. The *in vivo* recognition and diagnostic efficacy of o-DHLA si-Olfr2 NPs on AS plaques were evaluated in ApoE^-/-^ AS mice. Results indicated that o-DHLA si-Olfr2 NPs successfully visualized AS plaques, creating a distinct contrast compared to normal tissue. This nanoplatform demonstrates significant advantages in non-invasive diagnosis of AS.

In addition to the direct use of PA contrast agents, the visualization of O_2_⁻ in AS plaques also provides valuable insights into the vulnerability of plaques. Ma et al. developed a novel ratio-type semiconductor polymer nanoparticle (RSPN) for PAI of O_2_⁻ levels within AS plaques 110. The RSPN reacted with O_2_⁻, displaying enhanced photoacoustic signals at approximately 690 nm (with the signal at 800 nm serving as an internal reference). In *in vivo* experiments on plaque-bearing mice, the enhanced signals from RSPN were positively correlated with oxidative stress levels. Furthermore, in mice with plaque-associated pneumonia, the PA690/PA800 ratio was significantly higher compared to those with plaques alone. Histological analysis by H&E staining showed that plaques from pneumonia-bearing mice had thinner fibrous caps and necrotic cores, indicating greater plaque vulnerability. These findings suggest that RSPN can predict the vulnerability of AS plaques induced by pneumonia by assessing O_2_⁻ levels. This nanoparticle provides a non-invasive tool for the dynamic evaluation of oxidative stress levels in vulnerable plaques, and in the future, it may offer enhanced plaque assessment accuracy by combining with plaque-associated biomarkers.

Both fluorescent probes and contrast agents have shown excellent imaging capabilities for detecting CVDs. However, the sensitivity of these methods, such as the minimum detectable plaque content or the lowest number of targets, requires further experimental investigation. Current imaging approaches are based on ROS biomarkers associated with the pathological features of CVDs, but since ROS are not specific and can be overexpressed in other diseases, a deeper understanding of CVDs pathology and the identification of more specific biomarkers are essential to reduce misdiagnosis risks. Additionally, parallel detection of multiple biomarkers can help improve diagnostic accuracy. Multimodal imaging techniques offer a promising approach to enhance precision 111. It's also important to consider individual variations, as certain patients with underlying conditions or those on specific medications may influence the body's redox balance. This could alter the interaction between ROS-responsive materials and the body, potentially increasing the likelihood of misdiagnosis. Fluorescent probes and contrast agents typically reflect changes in one direction, either upregulation or downregulation. For instance, when ROS levels rise, ROS-responsive materials emit stronger signals. However, when treatment materials lower ROS levels, the signal enhancement may not decrease as expected 112. Thus, understanding the dynamic response of these materials and the impact of external factors is key to minimizing diagnostic errors. This is crucial for optimizing diagnostic accuracy and minimizing the risk of misdiagnosis in clinical applications.

### Treatment

CVDs encompass a variety of conditions, including thrombosis, AS, and MI, among others. While these diseases share common mechanisms, such as inflammation, oxidative stress, and elevated ROS levels, they also exhibit significant differences 113. For instance, MI is primarily associated with acute occlusion of the coronary arteries and subsequent ischemic damage, whereas AS involves lipid deposition, chronic inflammation, and plaque formation 114,115. Regardless of the underlying mechanisms, ROS play a crucial role in these conditions. Therefore, the design of ROS-responsive biomaterials tailored to different disease mechanisms holds significant clinical importance **(Table 1)**.

### Thrombotic diseases

Thrombi are primarily composed of activated platelets and fibrin, typically resulting from vascular injury, a hypercoagulable state, and slow blood flow 116. Thrombosis is a key mechanism in CVDs, leading to vascular obstruction, rapid interruption of blood supply, and resulting in ischemia, hypoxia, dysfunction, and potentially organ failure. Therefore, solving thrombotic diseases is crucial for improving patient outcomes in CVDs management, as effective treatment can restore blood flow, reduce inflammation, and prevent further cardiovascular complications.

In thrombotic diseases, endothelial cells mainly produce large amounts of ROS in response to stimuli such as ischemia and hypoxia. Elevated levels of ROS can cause oxidative stress damage to the endothelial lining and increase adhesion molecules, thereby exacerbating inflammation and promoting thrombosis 117. Thrombosis is characterized by elevated H_2_O_2_ levels and the accumulation of fibrin and platelets, providing critical insights for designing ROS-responsive biomaterials targeted at thrombosis 118. Thrombi associated with CVDs can be categorized into two types: those induced by implants and those resulting from the body's inflammatory response. The following sections will discuss the application of ROS-responsive biomaterials in each of these two types of thrombosis.

#### Anticoagulation

Cardiovascular implants, such as stents, heart valves, and pericardial tissue, play a critical role in the prevention and treatment of CVDs. However, these implants can trigger thrombosis after implantation, representing a significant complication in the management of coronary heart disease 119. During the implantation process, certain manipulations may cause local arterial injury, leading to the rupture of atherosclerotic plaques and damage to the intima or even the media of the vessel. This injury exposes procoagulant structures beneath the subendothelium, which release adhesion proteins such as von Willebrand factor that promote platelet adhesion, aggregation, and activation. Activated platelets release thromboxane A2 (TXA2), serotonin, adenosine diphosphate, and platelet factors, further enhancing platelet aggregation and thrombus formation 120. As a foreign material, the cationic charge on the surface of metal stents significantly increases platelet activation and the coagulation process. Platelets tend to deposit on the surfaces of metal stents, which diminish their biocompatibility and blood flow compatibility, thereby predisposing to in-stent thrombosis 121. Non-metallic implants primarily interact with soft tissues that resemble the mechanical properties of the cardiovascular system. As foreign materials, these tissues contain certain antigenic components which not only trigger acute and chronic inflammation but also cause heightened platelet activation, leading to diffusion, aggregation, and adhesion 122,123. When foreign substances are implanted into the cardiovascular system, a large number of inflammatory cells infiltrate the tissues to produce highly expressed ROS. These ROS can directly damage vascular endothelial cells and activate endothelial proinflammatory factors, such as interleukins and tumor necrosis factor, a process that further exacerbates local inflammatory responses. The inflammatory response, in turn, stimulates the continuous generation of ROS, creating a vicious cycle. To address the thrombosis associated with those implants, current treatments primarily involve drug therapy and coating techniques for the implant surface. However, drug therapy may lead to side effects such as bleeding, and the efficacy of coating techniques is limited by the sustained release and stability of the drugs. ROS-responsive biomaterials offer a promising solution by developing anticoagulant coatings on implant surfaces or crosslinking with materials such as pericardial tissue, which mitigate thrombosis occurrence.

The extracellular matrix (ECM) is a complex and dynamic network composed of macromolecular substances secreted by cells into the extracellular stroma, predominantly consisting of interstitial matrix and basement membrane. Inspired by its dynamic remodeling capabilities, Xiang et al. designed a biomimetic ECM bilayer nanostructure on the implant surface to confer long-term anti-inflammatory properties and reduce thrombus formation 124. The outer layer of the nanostructure was composed of polyvinyl alcohol (PVA) and poly(2-(4-((2,6-dimethoxy-4-methylphenoxy) methyl) phenyl)-4,4,5,5-tetramethyl-1,3,2-dioxab) (PBA), featuring ROS-responsive degradation characteristics. The inner layer consisted of PCL-PEG-PCL, Au-heparin nanoparticles, and indomethacin. In thrombus environments with high ROS expression, the outer PBA layer decomposed rapidly, thereby inhibiting acute inflammation. Concurrently, indomethacin in the inner layer effectively suppressed chronic inflammation and thrombosis through sustained release. Additionally, Au-heparin nanoparticles in the inner layer significantly enhance the long-term blood compatibility of the material. This study proposes a novel approach for designing long-term antithrombotic and anti-inflammatory implants with adaptive and self-regulating properties.

**Figure 7:**
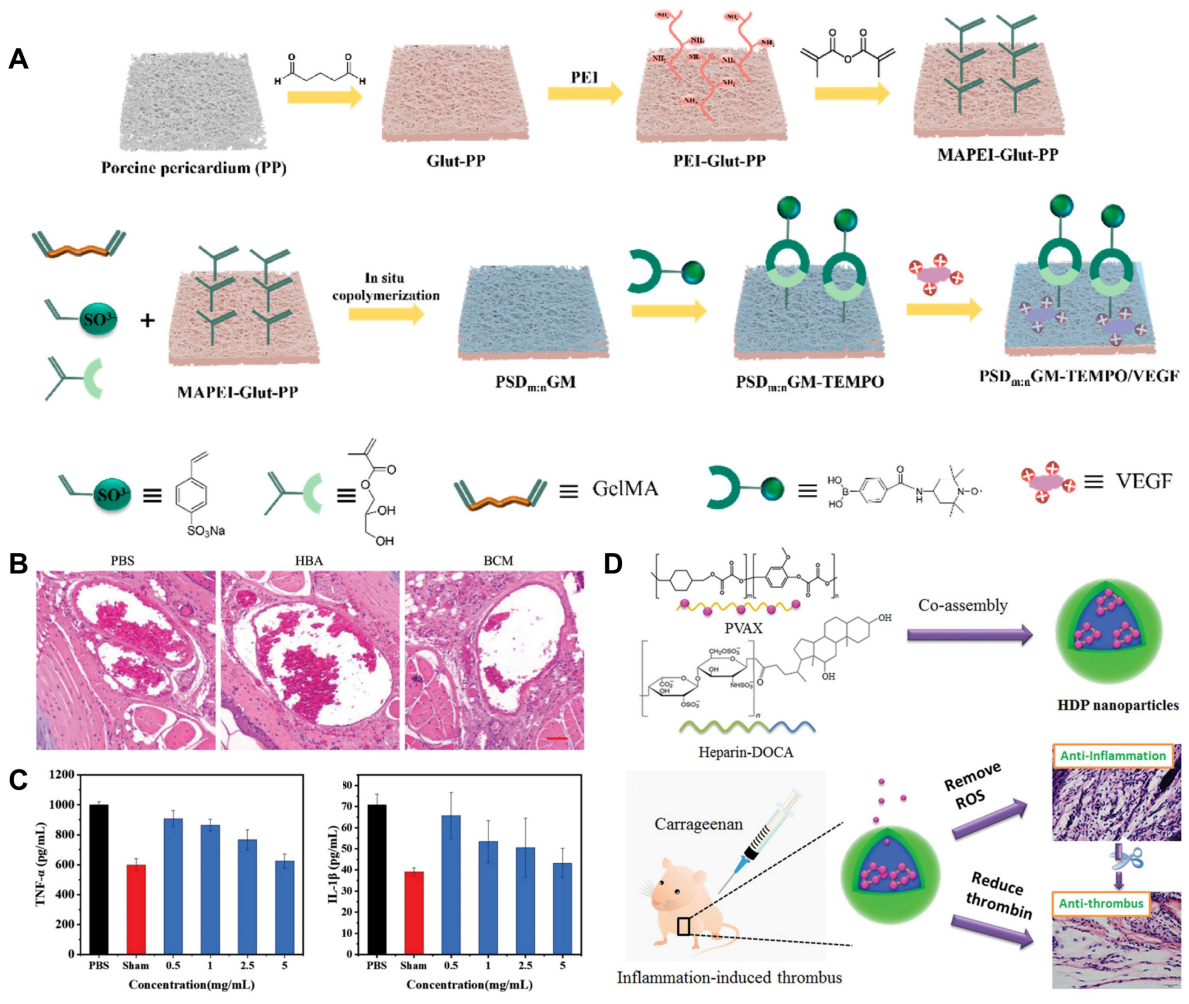
**(A)** Schematic illustrations of PSD_m:n_GM-TEMPO/VEGF preparation process. Adapted with permission from 126, copyright 2022, Elsevier Ltd. **(B)** BCM for the treatment of carrageenan-induced inflammation in mice. H&E staining of sections from the basal part of the black tail of mice.** (C)** Levels of TNF-α and IL-1β in the serum of each group of mice after the end of the experiment. Adapted with permission from 130, copyright 2023 Wiley-VCH GmbH. **(D)** Schematic illustration of nanoparticles effects on inhibition of thrombosis and inflammation. Adapted with permission from 131, copyright 2019, WILEY-VCH Verlag GmbH & Co. KGaA, Weinheim.

Some components of hydrogels, such as collagen and glycosaminoglycans, share similarities with ECM and can be used in implants to perform dynamic remodeling functions similar to those of the ECM. Xenogeneic pericardial tissue is frequently used in cardiac implants for treating CVDs; however, its inflammatory response and poor biocompatibility often lead to implantation failure 125. To improve this issue, Yang et al. developed a novel implant that utilized a hydrogel containing a high concentration of MMP degradation sequences to encapsulate the radical scavenger TEMPO **(Figure 7A)** 126. TEMPO was stabilized within the hydrogel through ROS-responsive boronate ester bonds. Additionally, they crosslinked a heparin-like polymer with the hydrogel on porcine pericardium and electrostatically loaded it with VEGF to enhance anticoagulant properties. In *in vivo* experiments, the new implant significantly reduced the expression of the pro-inflammatory factor tumor necrosis factor-alpha (TNF-α) and enhanced the expression of the anti-inflammatory factor IL-10 compared to porcine pericardium crosslinked solely with glutaraldehyde (Glut-PP). This study demonstrates that by crosslinking antioxidants, the novel vascular implant can effectively improve the inflammatory response of xenogeneic pericardial tissue in cardiovascular applications, significantly enhancing its biocompatibility and specifically addressing the issue of implantation failure.

The key to preventing and treating thrombosis induced by implants lies in reducing platelet adhesion and inflammation. ROS-responsive biomaterials introduced for this purpose must not only possess good antithrombotic properties and biocompatibility within the cardiovascular system but also establish a strong interface with the surface of the implant to ensure effective integration and functionality. The degradation time of materials plays a significant role in their ability to prevent thrombosis. Materials that degrade too quickly may lose their antithrombotic properties before sufficient healing occurs, while materials that degrade too slowly may increase the risk of long-term toxicity or unwanted side effects. Therefore, the degradation rate, effective duration, and long-term stability of the material must be carefully considered to ensure optimal antithrombotic function throughout the necessary treatment period.

#### Anti-inflammatory

Inflammation is a complex defense response of the body to endogenous or exogenous damaging agents. Recent scientific research has increasingly recognized that inflammation can induce thrombus formation. Inflammatory mediators such as lipopolysaccharides and cytokines have been reported to promote thrombosis. Tissue factor (TF) plays a critical role in linking the coagulation system with the inflammatory response; it is both an important coagulation factor and a molecular marker of endothelial injury 127. Under normal conditions, TF is present in adventitial cells of the vessel wall and does not come into contact with circulating blood. However, inflammatory mediators like lipopolysaccharides and cytokines can induce damage to endothelial cells and activate monocytes, leading to increased TF expression. Once TF is exposed to blood, it forms a FV-IIa complex, initiating the coagulation cascade 128. When cells are stimulated by inflammatory mediators, ROS (e.g., O_2_⁻, H_2_O_2_, ·OH) are produced by activation of NADPH oxidase, mitochondria, and other endothelial oxidases. Especially in the case of vascular endothelial injury, ROS generation is significantly enhanced. Therefore, under the inflammatory effect, ROS can promote the inflammatory response, increase platelet activation and promote the release of coagulation factors at the local injury site. The blood is often in a hypercoagulable state, which further promotes the formation of thrombosis. Due to their enhanced antithrombotic effects, currently used anticoagulants, such as heparin, often lead to destructive and potentially fatal side effects, including cerebral hemorrhage 129. DDS based on lipid and polymorphic designs frequently miss their targets or release heparin drugs prematurely during validation. Therefore, there is a need for ROS-responsive DDS that are suitable for the cardiovascular environment, which can extend the drug's lifespan in the bloodstream without altering its original biochemical properties. When designing ROS-responsive biomaterials, it is important to consider reducing inflammation while clearing thrombi. This design not only helps reduce the risk of thrombus recurrence but also offers new directions and strategies for improving cardiovascular treatments.

Self-assembling PEGylated poly(carbonate)s (BC) exhibits excellent biocompatibility and biodegradability, making it easy to form highly oxidation responsive micelles (BCMs). Chen et al. designed these oxidative-responsive micelles, which can rapidly release delivered drugs upon reacting with H_2_O_2_ and degrade to produce the anti-inflammatory small molecule, para-hydroxybenzyl alcohol (p-HBA) 130. In the mouse tail artery thrombosis model, different drugs were administered via tail vein injection. Subsequently, thrombus sections were prepared, showing that the BCM group had a significantly reduced thrombus volume in the vessel lumen compared to the PBS and HBA groups **(Figure 7B)**. At the end of the experiment, the levels of inflammatory cytokines in mouse serum were measured. The BCM micelles at 5 mg/mL exhibited the lowest inflammatory cytokine levels, similar to the normal group, further confirming their strong anti-inflammatory effect **(Figure 7C)**. Therefore, BCMs hold promise as a novel system for the rapid release of ROS-responsive drugs to treat inflammation-related thrombotic diseases. In another study, Xiang et al. developed ROS-responsive Hep-DOCA/PVAX (HDP) nanoparticles **(Figure 7D)** 131. The heparin conjugate Hep-DOCA reduced the amount of heparin needed, thereby minimizing potential side effects such as bleeding. PVAX was a copolymer of polyacid that contained vanillyl alcohol (VA), which served as an antioxidant to reduce inflammatory responses. VA was covalently linked to the PVAX backbone via H_2_O_2_-responsive oxalate ester bonds, allowing it to respond to ROS generation and release VA to exert anti-inflammatory effects. *In vitro* and *in vivo* experiments demonstrated that these nanoparticles possessed anti-inflammatory and anticoagulant properties, significantly improving treatment efficacy for inflammation-related thrombi while reducing the occurrence of side effects.

The treatment of inflammation and thrombotic complications requires an ideal DDS capable of addressing both inflammation and thrombus formation simultaneously. Indomethacin is a lipophilic anti-inflammatory drug, while nattokinase is a water-soluble fibrinolytic agent. To achieve combined drug delivery of these hydrophobic and hydrophilic agents, Xia et al. synthesized a polymer vesicle (IDM&NK@PPTV) containing amphiphilic blocks using reversible addition-fragmentation chain transfer polymerization, incorporating boronate ester bonds that respond to ROS 132. Indomethacin and nattokinase were loaded into these vesicles, allowing for effective release in mice, which demonstrated significant anti-inflammatory and antithrombotic effects. Experiments confirmed that this drug-loaded nano-micelle serves as an effective platform for precise drug release at sites of high ROS concentration, such as thrombi and inflamed tissues.

The application of ROS-responsive biomaterials in the treatment of inflammation-related thrombosis offers a unique and effective strategy. Inflammation and thrombosis are closely interconnected, with inflammatory factors such as cytokines and LPS activating TF in the coagulation system, thereby promoting thrombus formation. Meanwhile, oxidative stress and the excessive generation of ROS play a catalytic role in this process, further exacerbating thrombus development. Therefore, the role of ROS-responsive materials in this context goes beyond serving as carriers for therapeutic drugs; they can effectively suppress both inflammation and thrombosis formation by responding to the oxidative stress in the pathological microenvironment.

### Atherosclerosis

The formation and progression of AS is a complex and continuous process involving multiple key steps 133. Researches indicate that AS begins with endothelial injury, leading to the infiltration and accumulation of low-density lipoprotein (LDL) in the subendothelial space. Under pathological conditions, LDL is oxidized to oxidized LDL (ox-LDL), which activates endothelial cells, triggering an inflammatory response that promotes the expression of adhesion molecules and chemokines, thereby inducing the migration of inflammatory cells to the intima 134. Subsequently, monocytes differentiate into macrophages within the vascular intima. Macrophages engulf modified lipoproteins, forming foam cells, which are characteristic of early AS lesions. Foam cells, T lymphocytes, and mast cells accumulate in the intima, resulting in the production and release of various pro-inflammatory molecules and excess ROS. These molecules stimulate the proliferation of VSMC, which migrate to the intima and secrete ECM proteins, promoting the formation of fibrous plaques. As the lesion progresses, VSMC encapsulate the necrotic core rich in oxidized lipoproteins, forming a stable fibrous cap that ultimately leads to arterial hardening 135. Atherosclerotic plaques are unstable and prone to rupture. This can lead to the formation of blood clots, which block blood vessels, exacerbate the disease condition rapidly, increase the risk of acute cardiovascular events such as MI and pulmonary embolism, and pose a serious threat to life and health 136. Therefore, proactive treatment of AS can delay or halt the progression of CVDs, reduce the incidence and mortality of the disease, and improve patients' quality of life.

Traditional drug and surgical treatments for AS can suppress pathological progression to a certain degree, but they are accompanied by several drawbacks, such as low bioavailability, severe side effects, and poor surgical prognosis 137. The development of nanomaterials and hydrogels offers strategies to extend circulation time, dissolve drugs, and control drug release. AS is a chronic inflammatory disease, with oxidative stress as its core mechanism. Based on these findings, researchers have developed various ROS-responsive biomaterials. These materials, through antioxidant and anti-inflammatory effects, regulation of inflammatory cell activity, and reduction of plaque deposition, offer a potential solution for the treatment of AS.

#### Antioxidant and anti-inflammatory

CVDs are often associated with excessive ROS production, which further induces oxidative stress, leading to DNA damage, inflammation, and programmed cell death. Under oxidative stress, the pro-inflammatory I-κB kinase (IKK) is first activated, subsequently activating the inflammation-related transcription factor nuclear factor-κB (NF-κB), thus initiating the inflammatory response. The inflammatory response, in turn, accelerates ROS generation, creating a positive feedback loop. Studies show that inflammation and oxidative stress are critical factors in the exacerbation of CVDs such as, MI, IS, and MIRI. Therefore, antioxidant and anti-inflammatory therapies are considered effective strategies for CVDs, particularly AS. The use of ROS-responsive DDS to deliver antioxidant and anti-inflammatory drugs allows for precise targeting of atherosclerotic lesions, making it an important area of current research.

Andrographolide (Andro) exhibits strong anti-inflammatory activity by blocking the nuclear factor (NF) κB signaling pathway. However, its poor solubility limits its clinical applications. Nanoparticle DDS have significant advantages in enhancing the hydrophobic properties of drugs. In a study by Wu et al., amphiphilic diblock copolymer PEG-PPS was designed to form micelles for delivering Andro in the treatment of AS 138. Under the influence of excessive ROS in AS, PEG-PPS can transition from a hydrophobic to a hydrophilic state, enabling site-specific release of Andro. PEG-PPS consumed ROS at pathological sites, alleviating oxidative stress and thereby synergistically enhancing the therapeutic effects of Andro, offering an innovative strategy for AS treatment.

**Figure 8:**
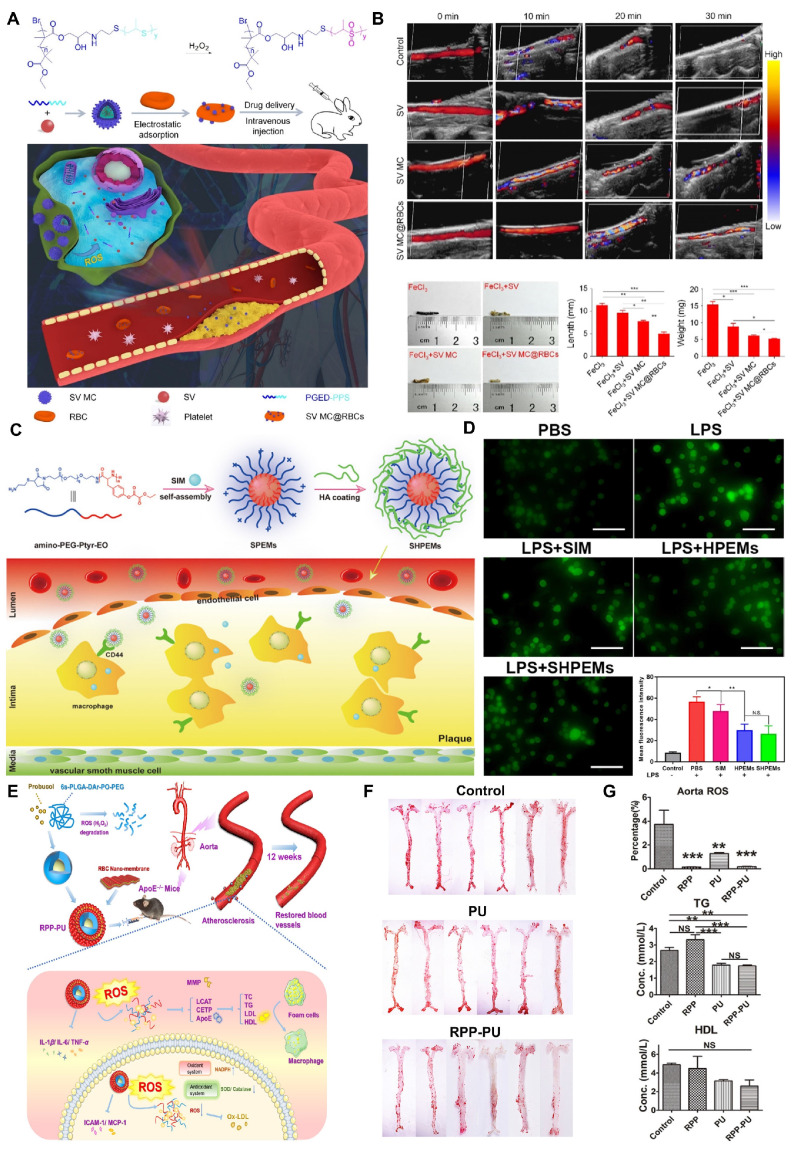
** (A)** Schematic illustration of SV MC@RBCs design and preparation. **(B)** Color-coded Doppler flow imaging was used to assess the degree of blockage in the rabbit carotid artery treated with FeCl_3_. Evaluation of the therapeutic effects of SV MC@RBCs on thrombus length and thrombus weight in the arterial vessel. Adapted with permission from 131, copyright 2021, Elsevier B.V. **(C)** The preparation route and targeted delivery process of SIM from the SHPEMs. **(D)** quantitative analyses for ROS production of LPS-induced RAW264.7 cells treated with PBS, SIM, HPEMs and SHPEMs by flow cytometry and cellular imaging. Scale bar: 100 μm. Adapted with permission from 140, copyright 2023, Springer Nature.** (E)** Preparation of RPP-PU and mechanism of improvements on atherosclerotic lesions. **(F)** Results of the entire aorta stained with oil red O. **(G)** Quantitative determination of ROS in the aorta by DCFH-DA. Serum levels of TG and HDL. Adapted with permission from 35, an open access article published 2023 by Chinese Pharmaceutical Association and Institute of Materia Medica, Chinese Academy of Medical Sciences under a CC-BY4.0 license.

In ROS-responsive DDS, nanoparticles benefit from passive targeting; however, they are prone to rapid clearance by the immune system, limiting their effectiveness for prolonged circulation. To evade immune clearance and enhance ROS treatment efficacy for AS, biomimetic strategies are particularly important. Shen et al. developed a smart system that responded to ROS and high shear stress, consisting of red blood cells (RBCs) and micelles loaded with the anti-inflammatory antioxidant simvastatin (SV) (SV MC)** (Figure 8A)** 139. The PPS in the micelles facilitated drug release in response to ROS and possessed in addition ROS-reducing properties, thereby enabling synergistic treatment with the drug and material. The incorporation of RBCs provided a protective layer for SVMC, reducing rapid clearance and prolonging circulation time. Notably, this study developed a shear-sensitive DDS that utilized the high shear stress induced by AS-related vascular narrowing, offering a non-invasive approach for treating vascular diseases. In the FeCl_3_-induced rabbit carotid artery thrombosis model, color-coded Doppler flow imaging showed no blood flow interruption at 10 minutes and the strongest flow signal at 30 minutes in the SV MC@RBC group, indicating a significant delay in arteriosclerosis progression. Thrombus length measurements revealed that SV MC@RBCs resulted in the shortest thrombus length and lowest weight, demonstrating the efficacy of SV MC in inhibiting plaque thrombus formation. **(Figure 8B)**. In addition to targeting high shear stress, receptors on macrophage surfaces present another effective target. Mu et al. designed HA-coated micelles loaded with simvastatin (SIM) **(Figure 8C)** 140. The HA coating effectively targeted CD44-positive inflammatory macrophages. The micelles were constructed from poly (ethylene glycol)-poly (tyrosine-ethyl oxalate) (PEG-Ptyr-EO), where the oxalate bonds could be oxidized by H_2_O_2_ to alcohol and CO_2._ This process consumed ROS at pathological sites, inhibited the accumulation of pro-inflammatory macrophages, and alleviated oxidative stress. In cellular experiments, to investigate the antioxidant effect of SHPEMs, DCFH-DA was used as a probe to assess cells treated with different groups. From the DCF fluorescence images in **Figure 8D**, a significant reduction in ROS levels was observed, indicating that SHPEMs could effectively scavenge intracellular ROS. In addition, *in vivo* studies were carried out to evaluate the therapeutic effects. After 8 weeks of high-fat diet feeding, aortas from ApoE^-/-^ mice were isolated, and plaque area was measured. The SHPEMs group showed significantly smaller lesion areas in comparison with other groups, indicating a remarkable enhancement in AS effects. In a study by Liang et al., a nano-biomimetic system (RPP-PU) was designed, consisting of red blood cell membrane (RBCM) wrapped nanoparticles loaded with the drug probucol (PU) using a polylactic-co-glycolic acid-phenyl ring-peroxy oxalate bond-polyethylene glycol (6s-PLGA-DAr-PO-PEG) **(Figure 8E)** 35. The conjugation of the phenyl ring with peroxy oxalate enhanced the efficient removal of H_2_O_2_ in the atherosclerotic microenvironment. In* in vivo* experiments with ApoE^-/-^ mice, it was demonstrated that RPP-PU effectively reduced ROS levels in the aorta. At the same time, PU also had a strong lipid-lowering effect, primarily by reducing LDL levels and enhancing the reverse transport of HDL. The lipid-lowering effect of the RPP-PU group was significantly better than that of the free PU group **(Figure 8F)**. To further evaluate the lipid-lowering effect of the material, researchers isolated and froze the serum, and results revealed that the RPP-PU treatment group significantly lowered serum lipid levels **(Figure 8G)**. The excellent stability and sustained release properties of this nanomaterial allow for prolonged ROS clearance and regulation of lipid metabolism. To achieve more efficient targeting, researchers are moving beyond reliance on a few naturally occurring surface proteins and are using genetic engineering to express target proteins. Zhong et al. edited endothelial cells to overexpress the VLA-4 surface protein, which was then encapsulated in RAP nanoparticles to create a nanotherapeutic agent (OEM@RAP NPs) 141. VLA-4 can specifically bind to the overexpressed receptor VCAM-1 on inflammatory cells, enabling targeted delivery of drugs to atherosclerotic lesions. Additionally, it allowed for precise release of RAP in response to ROS. This biomimetic DDS holds promise for inhibiting the progression of AS.

The combination of specific diagnosis and treatment of AS is crucial for identifying and accurately treating plaque sites. Ma et al. developed a biomimetic nanoparticle platform (RBC/LFP@PMMP) that combined lipid-specific imaging with a ROS-responsive prodrug polymer, wrapped in RBCM for the diagnosis and treatment of AS. In circulation, LFP exhibited red fluorescence, allowing for real-time tracking of RBC/LFP@PMMP 142. Upon reaching atherosclerotic lesions, the oxalate bonds in RBC/LFP@PMMP degraded in response to ROS, releasing LFP. LFP specifically bound to lipids and emitted bright green fluorescence, allowing lesion labeling and imaging. Additionally, the degradation process released the potent anti-inflammatory drug Pred, providing targeted treatment at the lesion site. This biomimetic nanoparticle platform integrates diagnostic and therapeutic functions, offering a promising strategy for the detection and treatment of AS.

Antioxidant and anti-inflammatory therapy are crucial in treating AS, as excessive ROS production triggers oxidative stress and inflammation, thus leading to vascular damage and plaque formation. Studies indicate that antioxidant and anti-inflammatory treatments can effectively mitigate these effects and slow down the progression of AS. ROS-responsive DDS enable precise targeting of lesions, alleviating oxidative stress and inflammation to achieve superior therapeutic outcomes. Nanomaterials with antioxidant properties can locally deplete ROS and regulate vascular inflammation. Additionally, strategies such as genetic engineering or receptor binding can enhance targeting and therapy efficacy. These innovative approaches offer promising solutions for more effective and long-term AS treatment.

#### Regulate macrophages

Macrophages play a dual role in AS, primarily through the contrasting functions of pro-inflammatory M1 and anti-inflammatory M2 macrophages. M1 macrophages phagocytize ox-LDL to form foam cells, which are key components of atherosclerotic plaques and accumulate within the arterial walls to create the necrotic core. Pro-inflammatory factors released by M1 macrophages, such as TNF-α, interleukin-6, and IL-1β, promote inflammatory cell infiltration and endothelial damage, weaken the fibrous cap of plaques, and elevate the risk of plaque rupture, thereby worsening the progression of AS 143. In contrast, M2 macrophages have anti-inflammatory properties, secreting anti-inflammatory cytokines that promote angiogenesis and healing of damaged myocardium 144. Therefore, promoting the transition of macrophages from the M1 to the M2 phenotype is a crucial strategy for treating AS, achievable by reducing local oxidative stress or modulating signaling pathways such as NF-κB. ROS-responsive biomaterials can serve as drug carriers to target the delivery of agents that induce macrophage polarization, thus facilitating the shift to the M2 phenotype. Additionally, reducing the number of activated macrophages within plaques is another viable approach.

Discoidal reconstituted high-density lipoprotein (d-rHDL) promotes cellular cholesterol efflux and targets the delivery of anti-atherosclerotic drugs. Zhang et al. utilized d-rHDL for the delivery of anti-atherosclerotic drugs, modifying it with ROS-responsive materials to create d-rHDL modified with a polyethylene glycol-based Fc/β-CD supramolecular copolymer (PF/TC) 145. ROS can efficiently dissociate the β-CD/Fc copolymer by oxidizing hydrophobic Fc to hydrophilic Fc^+^, thereby releasing the encapsulated drug atorvastatin. Atorvastatin not only mediated intracellular cholesterol efflux but also promoted the M2 polarization of macrophages, exerting a dual anti-inflammatory effect.

**Figure 9:**
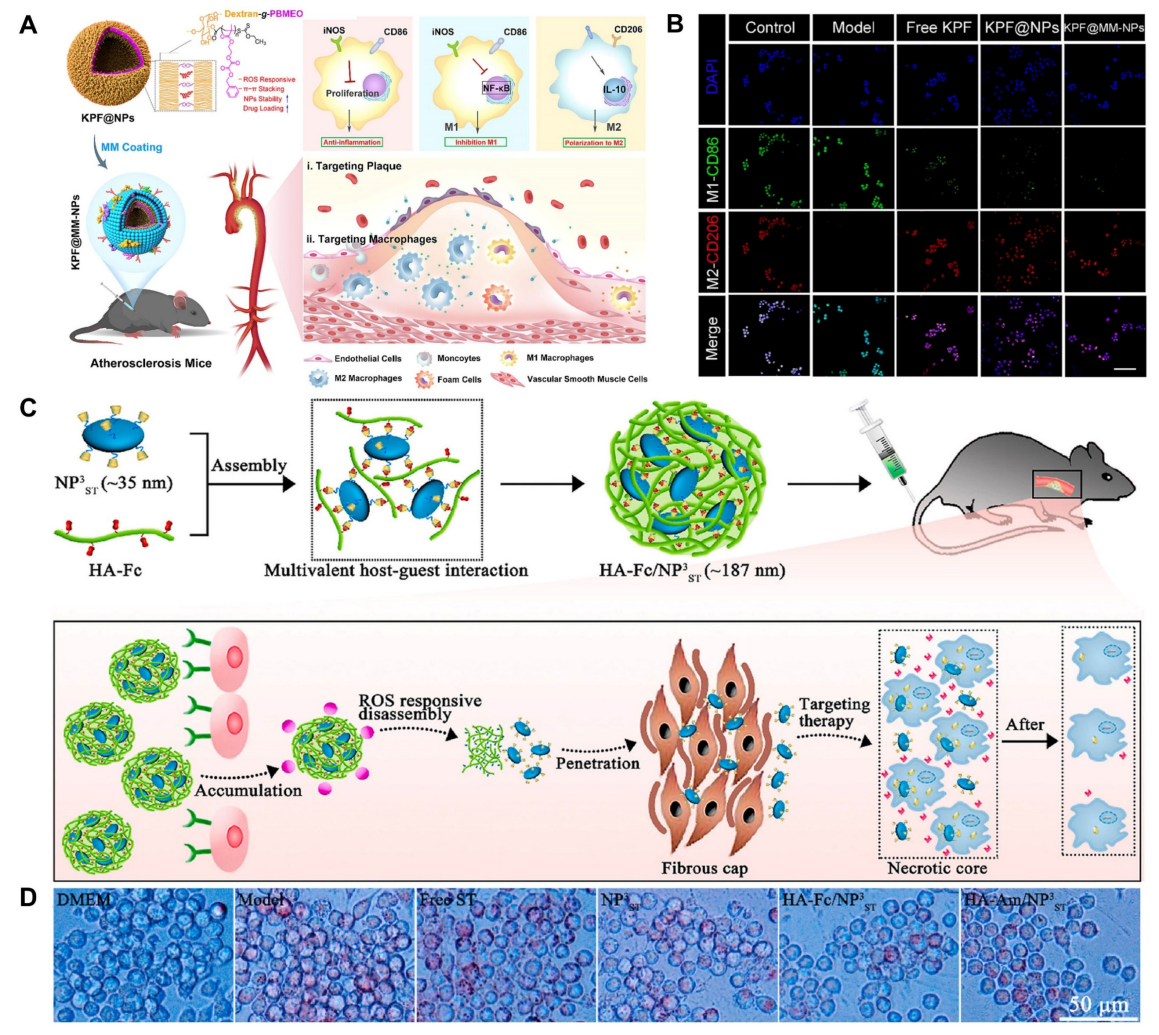
**(A)** Preparation of KPF@MM-NPs as novel KPF tool and i.p.-administrated for macrophage-targeted delivery in AS plaque. **(B)** Representative immunofluorescence stains of M1 (FITC-CD86, green) and M2 (F555-CD206, red) macrophages re-polarization after free KPF, KPF@NPs and KPF@MM-NPs treatment. Nuclei were stained with DAPI (blue). Scale bar: 50 μm. Adapted with permission from 146, copyright 2022, Elsevier B.V. **(C)** Illustration of the assembly of ROS-responsive size-reducible HA-Fc/NP^3^_ST_ nanoassemblies and their desired performance *in vivo*. **(D)** Oil red O staining of intracellular lipid deposition imaged by inverted fluorescence microscope. Adapted with permission from 29, an open access article published 2023 by Elsevier B.V under a CC-BY4.0 license.

Drugs loaded in biomimetic macrophage membranes (MM) offer significant advantages in treating inflammatory diseases, as they can evade clearance by the reticuloendothelial system and their membrane-specific receptors can be recognized by inflammatory factors in the environment, enabling targeted drug delivery. Zhao et al. developed macrophage membrane-coated nanoparticles (KPF@MM-NPs) loaded with kaempferol (KPF), where dextran-polymer of bis(methoxyethoxymethyl) ether oxide (PBMEO) nanoparticles triggered ROS-responsive release of KPF at pathological AS sites** (Figure 9A)** 146. Research indicated that KPF can downregulate the ROS-activated NF-κB pathway, inhibited the expression of pro-inflammatory cytokines, and promoted the repolarization of M1 macrophages to the M2 phenotype, thereby achieving therapeutic effects in AS. To demonstrate that KPF@MM-NPs promote M2 macrophage polarization and reduce M1 macrophages levels, RAW264.7 cells pre-cultured with LPS (1 μg/mL) were treated with different KPF samples and analyzed using immunofluorescence. As shown in **Figure 9B**, CD206, a marker for anti-inflammatory M2 macrophages, was upregulated in the KPF-treated groups. Quantification of the M2/M1 ratio revealed that KPF@MM-NPs exhibited the highest polarization level, approximately 2.5 times higher than that of the free KPF group. This indicates that incorporating KPF into the nanoscale platform enhances its therapeutic efficacy. M1 macrophages, serving as cellular effectors in inflammation and tissue repair, can actively target inflamed vasculature via integrins, making them highly promising for targeted therapy in AS. Qu et al. developed an RVT prodrug using 3-nitrophenylboronic acid (PBA) and camouflaged the prodrug with MM (MM@CD-PBA-RVT) 147. Upon stimulation by pathological ROS, the prodrug was triggered to release through the cleavage of the covalent bond between carbon and boron in PBA. Both *in vivo* and *in vitro* experiments demonstrated that MM@CD-PBA-RVT effectively promoted the transition of macrophages from the M1 to the M2 phenotype, thereby alleviating inflammation and facilitating the regression of AS plaques. This nanoparticle, using MM for targeting, also represents a promising and feasible strategy for developing effective and safe AS management approaches. Active macrophages within plaques are considered a factor in plaque instability and play a crucial role in the progression of AS. Therefore, Kim et al. proposed a method to selectively detect and eliminate plaque-associated macrophages by developing a ROS-responsive photodynamic therapy (PDT) drug (MacTNP) conjugated with R-Chlorin e6 and HA 148. Activated macrophages contain high levels of ROS, particularly peroxynitrite. When macrophages internalized MacTNP, ROS cleaved the HA, leading to the degradation of the nanoparticles. Specifically, peroxynitrite induced the selective degradation of HA at the N-acetylglucosamine β-(1→4) glycosidic bond. The processed MacTNP emitted near-infrared fluorescence within activated macrophages, inducing the photosensitizer to generate singlet oxygen that killed the macrophages. Compared to human dermal fibroblasts and non-activated macrophages, the ROS-responsive MacTNP demonstrates stronger phototoxicity, showing great potential for selective NIR fluorescence imaging and PDT *in vivo* for atherosclerotic lesions with a high target-to-background ratio.

Macrophages play an important role in the inflammatory response, lipid accumulation and plaque formation in AS, so regulating their function has become a new strategy for the treatment of AS. Future studies can focus on regulating the phenotypic switching of macrophages through drugs or nanomaterials to reduce inflammation, inhibit plaque development, and promote vascular repair. In addition, targeted delivery systems can precisely deliver therapeutic agents to macrophages, improving therapeutic efficacy and reducing side effects. However, how to balance the functions of macrophages to avoid excessive suppression of immune responses remains a key challenge for future research.

#### Clearance of lipids

AS plaques have a complex structure, primarily consisting of a lipid core, foam cells, a fibrous cap, and ECM components. Among these, oxLDL is the main form of lipid accumulation in plaques, triggering inflammatory responses and attracting immune cells. The rupture or erosion of unstable plaques can lead to acute thrombosis, resulting in serious cardiovascular events such as acute coronary syndrome 149. To treat AS, researchers primarily focus on enhancing cholesterol efflux or inhibiting the function of the mammalian target of RAP (mTOR) at the genetic level to reduce lipid deposition.

Salvia miltiorrhiza extracts have been shown to be effective against AS. Among these, tanshinone IIA (TS-IIA) is a lipophilic compound that inhibits LDL oxidation and foam cell formation, while salvianolic acid A (SAA) is a hydrophilic component that promotes cholesterol efflux and reduces oxidative stress. Tang et al. developed a ROS-responsive prodrug micelle (TS-IIA-PM) by self-assembling an amphiphilic block copolymer PEG5000-SAA/PLA10000-APBA, successfully combining the poorly soluble TS-IIA and SAA 150. After incubating the micelle in 1 mM H_2_O_2_ for 48 hours, the borate ester bonds cleaved, releasing the active drugs. In *in vivo* experiments, both compounds exhibited a strong synergistic effect against AS at low doses.

Nanoparticles must penetrate the fibrous cap to reach foam cells and exert their therapeutic effects. To address the limited permeability of deep atherosclerotic plaques, He et al. developed a ROS-responsive, size-reducible nanoparticle component (HA-Fc/ NP^3^_ST_) **(Figure 9C)** 29. The conjugate of HA and Fc was cross-linked with NP^3^_ST_, forming HA-Fc/NP^3^_ST_. Upon ROS oxidation in the plaque area, it degraded, releasing NP^3^_ST_, which could penetrate deeply into the plaque and effectively permeate macrophage spheres, enhancing cholesterol efflux and exerting anti-inflammatory effects. In the* in vitro* anti-AS experiment, Oil Red O staining, a common method for visualizing lipid deposition, was employed to evaluate intracellular lipid accumulation. This demonstrated that the nanoparticles effectively reduced lipid accumulation, which was closely correlated with their capacity to promote cholesterol efflux **(Figure 9D)**.

mTOR, which regulates autophagy and lipid metabolism, is crucial for the onset and progression of AS. Thus, designing effective materials to silence mTOR holds promise for efficiently treating AS. Gao et al. developed an RNA interference antisense oligonucleotide (ASO) delivery platform based on cerium oxide nanowires and a plaque-penetrating peptide (S2P) 151. In the presence of H_2_O_2_, the platform released the loaded ASO around atherosclerotic plaques due to the strong binding affinity between H_2_O_2_ and Ce^4+^. The ASO targeted mTOR to inhibit its function, activated autophagy, prevented foam cell formation, and reduced lipid accumulation, thereby slowing the progression of AS. This targeted platform reduces the deposition of atherosclerotic plaques at the genetic level.

Lipid clearance is a critical strategy for the treatment of AS, primarily by reducing the accumulation of oxLDL and promoting cholesterol efflux to alleviate plaque formation. ROS-responsive DDS allow site-specific drug release at AS lesions, penetrating the fibrous cap of plaques to facilitate drug infiltration into foam cells, enhancing anti-inflammatory and antioxidant effects. For instance, targeting mTOR to control autophagy and lipid metabolism can reduce lipid deposition and inhibit foam cell formation. ROS-responsive nanoparticles not only improve drug precision and penetration but also regulate local oxidative stress, promoting cholesterol clearance and slowing AS progression. Combined with these mechanisms, ROS-responsive therapeutic platforms offer a new direction for precise treatment of AS.

### Myocardial infarction

MI is a serious cardiovascular condition caused by the rupture or erosion of unstable coronary plaques, which triggers thrombosis and leads to sustained and complete occlusion of the coronary arteries. Following vascular occlusion, the affected myocardial region undergoes coagulative necrosis, accompanied by interstitial edema and infiltration of inflammatory cells. During myocardial injury, angiogenesis, fibrotic scar formation, and inflammatory responses occur simultaneously 152. In the early stages of MI, oxidative stress, myocardial cell apoptosis and necrosis, collagen deposition, and scar tissue formation lead to myocardial damage, reducing cardiac contractile and diastolic function. In the later stages of MI, the myocardium experiences pathological hypertrophy, leading to ventricular remodeling. This includes increased left ventricular volume, thinning of the infarcted myocardium, and thickening of the non-infarcted myocardium. These changes disrupt ventricular contraction and electrical activity, ultimately resulting in heart failure 153. Consequently, aggressive treatment of MI is capable of reducing the death of myocardial cells, safeguarding cardiac function, and mitigating the risk of exacerbation of CVDs. Although current treatments for MI include pharmacological therapy and PCI, these approaches do not address the repair of infarcted myocardial tissue 154. ROS-responsive materials, which can modulate the local redox environment and regulate the release of therapeutic agents, offer potential in promoting tissue repair and mitigating oxidative stress in the damaged heart. Given the crucial role of oxidative stress and ROS in both early and late stages of MI, targeting ROS production through responsive biomaterials could provide a promising strategy for managing myocardial injury, reducing inflammation, and preventing long-term remodeling. In both early and late stages of MI, inflammatory responses and oxidative stress generate substantial amounts of ROS, which are involved in myocardial cell apoptosis and ventricular remodeling. Therefore, designing ROS-responsive biomaterials tailored to the pathological processes at different stages of MI may enable more effective treatment.

#### Anti-inflammatory and anti-oxidative

Anti-inflammatory and antioxidant strategies are crucial for treating MI. During MI, myocardial cells are damaged and necrotic due to ischemia and hypoxias, leading to increased production of ROS. Excessive ROS production, coupled with an imbalance in the antioxidant system, triggers oxidative stress, exacerbating cell apoptosis and inflammatory responses 155. Excessive inflammation, in turn, increases ROS production, further damaging tissues and promoting cardiac structural remodeling, ultimately leading to heart dysfunction and heart failure 156. Therefore, alleviating oxidative stress in the early stages of MI helps reduce cell death and inflammatory responses.

Cardiac patches exhibit excellent biocompatibility, providing mechanical support that promotes the attachment, proliferation, migration, and differentiation of myocardial cells. Building on previous research, Xie et al. developed a novel PFTU with strong ROS-scavenging capabilities, electrospinning it into a composite ROS-responsive cardiac patch PFTU/gelatin (PFTU/GT) 157. This material leveraged the decomposition characteristics of metal-thioether linkages in response to ROS, acting directly as an antioxidant to protect cells from oxidative stress, thereby reducing the inflammatory responses after MI. Experimental results indicated that implantation of the PFTU/GT fiber patch significantly reduced ROS levels and lipid peroxidation in rats with MI compared to the control group without the patch. This study combines the mechanical support of PUTK with its ROS responsiveness, presenting promising applications for the treatment of MI. Functional cardiac patches play a crucial role in cardiac function reconstruction following MI, and elastic fiber patches can effectively match the cyclic contractions of the heart, providing prolonged mechanical support. Yao et al. synthesized a ROS-responsive biodegradable elastic PUTK to create fiber patches loaded with methylprednisolone (MP) for treating MI 32. Research indicated that during a treatment period of up to 28 days, the PUTK/MP fiber patches significantly promoted the recovery of cardiac function and effectively reduced myocardial fibrosis and adverse cardiac remodeling.

**Figure 10:**
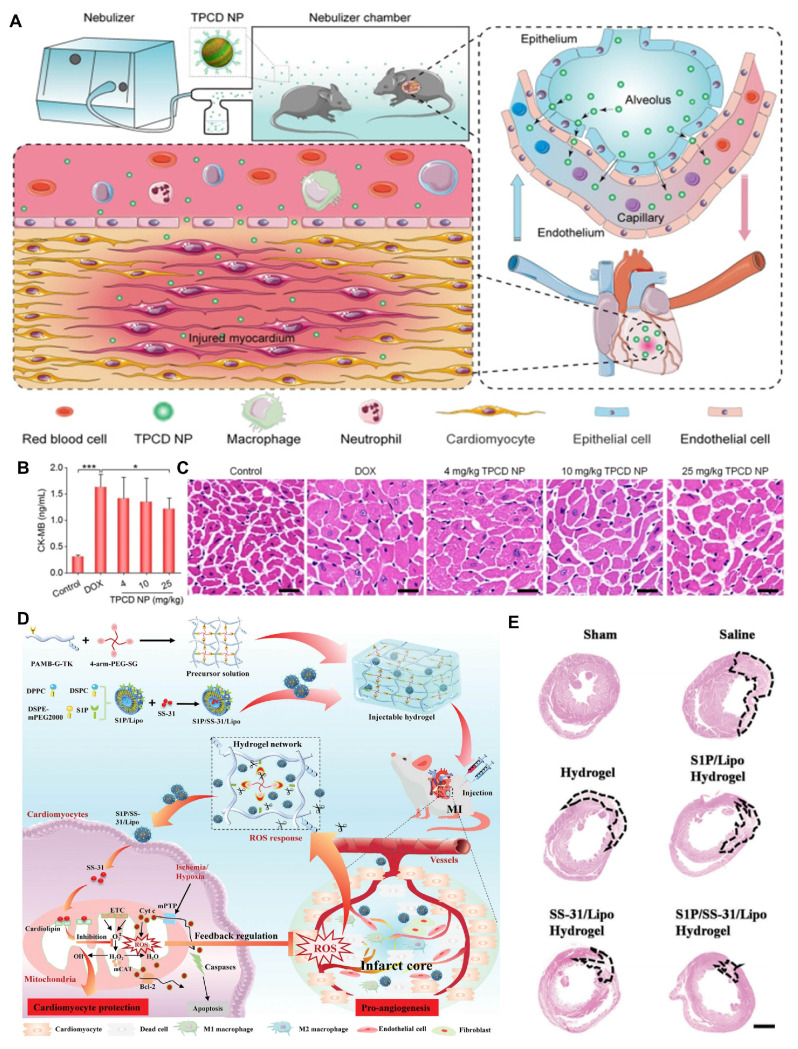
**(A)** Schematic illustration of *in vivo* targeting of the injured myocardium by inhalation of a nano-therapy TPCD NP.** (B)** Serum levels of CK-MB. **(C)** H&E-stained histological sections of hearts. Control, healthy mice treated with saline; DOX, mice treated with DOX and saline. In different TPCD NP groups, diseased mice were treated with different doses of TPCD NP. Adapted with permission from 158, an open access article published 2021 by Ivy Spring under a CC-BY4.0 license. **(D)** Schematic illustration of the formation and mechanism of an S1P/SS-31/Lipo-encapsulated ROS-responsive composite hydrogel for the efficient treatment of MI. **(E)** H&E staining of the myocardial border and infarcted areas (scale bars: 2 mm). Adapted with permission from 159, copyright 2021, Ivy Spring.

Another study developed ROS-responsive biomaterials that function directly as antioxidants. Liu et al. synthesized ROS-scavenging materials known as TPCD-NPs, composed of sequentially conjugated Tempol and 4-(hydroxymethyl) phenylboronic acid** (Figure 10A)** 158. To enhance therapeutic efficacy, TPCD-NPs were loaded with the anti-inflammatory drug Ac2-26, referred to as ATPCD NPs. In the DOX-induced heart failure mouse model, serum CK-MB (a cardiac injury biomarker) levels were quantitatively measured, confirming that 25 mg/kg TPCD NP effectively inhibited myocardial damage in the mice **(Figure 10B)**. Additionally, H&E-stained heart tissue sections revealed a significant reduction in pathological abnormalities such as cytoplasmic vacuolation and nuclear swelling in myocardial cells, especially after treatment with 25 mg/kg TPCD NP **(Figure 10C)**. Via inhalation, the nanoparticles can cross the pulmonary epithelium and endothelial barriers, accumulating in the heart through transcellular and paracellular routes, demonstrating an innovative pulmonary circulation-mediated cardiac targeting strategy.

Myocardial cells require a certain level of ROS to maintain normal function; however, some ROS scavengers may fail to achieve the desired therapeutic effects due to excessive depletion of normal ROS levels. Elamipretide (SS-31) is a mitochondrial-targeted antioxidant peptide that primarily prevents ROS-mediated cardiolipin oxidation by binding to cardiolipin. This antioxidant peptide selectively targets damaged mitochondria, reducing ROS-induced mitochondrial damage while maintaining the ROS levels necessary for normal myocardial cell function. Zheng et al. developed a ROS-responsive PAMB-G-TK/4-arm-PEG-SG hydrogel for localized drug delivery 159. SS-31 and the pro-angiogenic factor S1P were encapsulated in liposomes to form S1P/SS-31/Lipo nanoparticles** (Figure 10D)**. This hydrogel was cross-linked via thioketal links, allowing it to undergo deformation in the presence of excess ROS during MI, rapidly releasing the encapsulated S1P/SS-31/Lipo for localized treatment of MI. In a rat MI model, the liposome-composite hydrogel significantly improved cardiac function by clearing excess ROS, enhancing mitochondrial dysfunction, and promoting angiogenesis. The *in vivo* therapeutic efficacy of the material was tested in SD rats. H&E staining was used to assess collagen deposition and identify fibrotic areas following MI. The treatment group with S1P/SS-31/Lipo-loaded conductive hydrogel demonstrated strong inhibition of fibrosis **(Figure 10E)**.

In MI, oxidative stress and inflammation mutually exacerbate myocardial damage. Various ROS-responsive materials, such as PFTU/GT cardiac patches and PUTK/MP fiber patches, have shown promise in improving heart function by clearing ROS and reducing inflammation. Based on this, researchers can explore the development of multi-modal ROS-responsive smart material systems. For example, nano-composites that respond to ROS concentration, temperature, and specific myocardial biomarkers could be developed. These systems would enable precise sensing of MI microenvironment changes, allowing for programmed, on-demand drug release. Initially, antioxidants could neutralize excess ROS, followed by the release of anti-inflammatory and repair-promoting factors for staged treatment. Furthermore, combining ROS-responsive elements with gene editing technologies could trigger gene expression regulation, enhancing myocardial cells' intrinsic antioxidant and anti-inflammatory abilities, offering an innovative multi-dimensional approach for MI treatment.

#### Anti-apoptosis

After MI, excessive ROS produced by the heart can cause oxidative damage to DNA, leading to early apoptosis of myocardial cells. Additionally, the accumulation of ROS causes mitochondrial dysfunction, reducing ATP synthesis and metabolic regulation, which promotes the leakage of apoptotic factors and accelerates myocardial cell death 160. Therefore, designing biomaterials that can inhibit apoptosis is crucial for treating MI.

**Figure 11:**
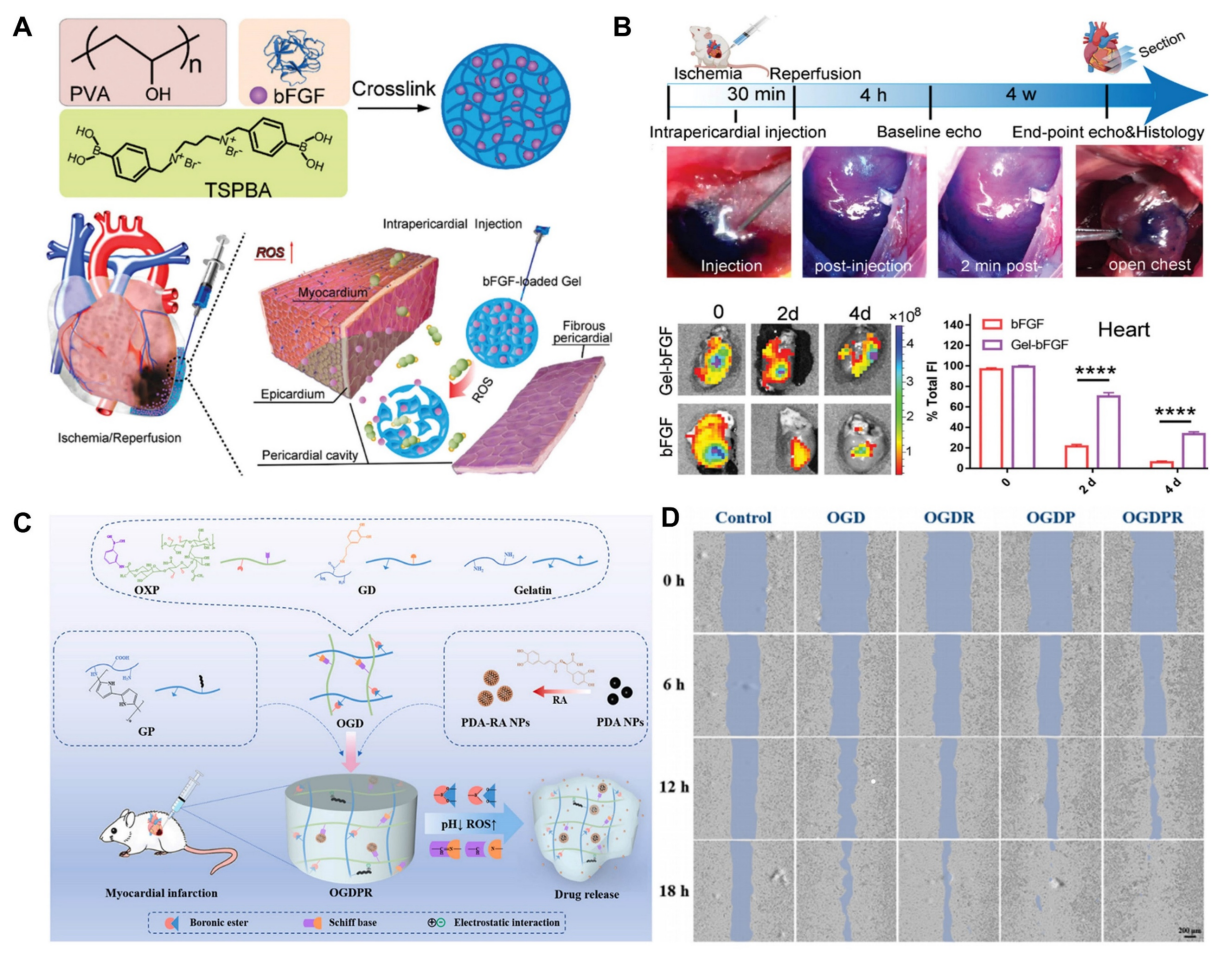
** (A)** Schematic illustration of Gel-bFGF fabrication and overall strategy. **(B)** Experimental timeline of animal studies; Representative images captured during the injection process; *Ex vivo* IVIS imaging of heart tissues following intrapericardial injection of bFGF alone or Gel-bFGF at baseline, 2 days, and 4 days post-injection. Quantification of fluorescence intensities of bFGF within the heart tissues. Adapted with permission from 161, copyright 2021, Wiley-VCH GmbH. **(C)** Schematic illustration of the preparation of a pH/ROS dual-responsive conductive hydrogel encapsulating Polydopamine (PDA)-RA NPs and its use in MI treatment. **(D)** Representative images of HUVECs cultured with different hydrogel extracts for 0, 6, 12, and 18 h. Scale bar: 200 μm. Adapted with permission from 163, an open access article published 2023 by Elsevier B.V under a CC-BY4.0 license.

In recent years, basic fibroblast growth factor (bFGF) has been shown to promote cardiac repair through anti-apoptotic and pro-angiogenic mechanisms. bFGF requires a safe and effective drug delivery strategy to ensure sustained and controlled release for MI treatment. Current drug delivery methods include intramuscular injection, intracoronary injection, intravenous injection, and placement of epicardial cardiac patches. However, these methods have limitations, such as low retention rates and significant trauma from open-heart surgery. To address these limitations, Li et al. proposed a novel drug delivery method: direct injection of hydrogel into the pericardial cavity (i.e., intrapericardial injection or iPC injection) **(Figure 11A)** 161. They synthesized a ROS-responsive crosslinked PVA hydrogel and loaded it with bFGF for myocardial repair. This injection method allowed the hydrogel to spread over the epicardial surface, which facilitated bFGF to penetrate the endocardium and bind to the myocardium. In the animal model, after the injection of iPC hydrogel, the biodistribution of Gel-bFGF was assessed in rats. Compared to the saline group, the ROS-responsive hydrogel enhanced the retention of bFGF in the heart** (Figure 11B)**. This indicated that iPC was an effective drug delivery method, and its accumulation at the injury site enhanced therapeutic action of bFGF. The investigators finally demonstrated the feasibility of performing iPC pathways in human patients undergoing standard LARIAT surgery, highlighting the value and potential for clinical translation. Xiang et al. prepared a ROS-responsive nanoparticle PEG-PPS-PEG@MR409 NPs with growth hormone releasing hormone agonist (MR409) loaded as a hydrophilic peptide in the vesicle core 162. By fluorescently labeling MR409, PEG-PPS-PEG@MR409 NPs were found to be efficiently internalized by cardiomyocytes. MR409, which was released in response to ROS in cardiomyocytes, had a significant effect on preventing cardiomyocyte apoptosis. The mouse model of MI showed a significant reduction in infarct size at 28 days after treatment with PEG-PPS-PEG@MR409 NPs. In the *in vivo* targeting experiments, the fluorescence intensity of the liver and kidney was higher in MI and non-MI mice, although the drug concentration at the lesion site was increased, there was still room for improvement to reduce the metabolism of PEG-PPS-PEG@MR409 NPs in the liver and kidney.

Conductive hydrogels show great potential for MI treatment due to their ability to restore electrical transmission in infarcted areas and promote synchronized contraction of myocardial cells. Zhang et al. developed a pH/ROS dual-responsive injectable hydrogel (OGDPR) **(Figure 11C)** 163. The borate ester bonds in the hydrogel responded to ROS, enabling on-demand release of the drug rosmarinic acid (RA). RA scavenged ROS and inhibited apoptosis, thereby reducing myocardial fibrosis. Polypyrrole, as the conductive polymer in OGDPR, effectively imparted conductivity to the hydrogel and recruited endothelial cells to promote neovascularization. In cell experiments, the effect of the hydrogel on endothelial cells was evaluated through a scratch assay. The results showed that after 16 hours, cell migration was observed at all scratch sites in the OGDPR group. This indicated that, compared to the control and other groups, OGDPR significantly promoted endothelial cell migration, which benefited tissue regeneration and repair **(Figure 11D)**. OGDPR exhibits therapeutic efficacy at various stages of inflammation, proliferation, and fibrotic remodeling after MI, offering better treatment prospects for MI patients.

Hydrogen sulfide (H_2_S), an important biological signaling molecule, plays a role in various physiological and pathological processes. H_2_S is reported to play a critical role in regulating cardiovascular function and is considered a promising therapeutic agent for MI. However, H_2_S is a gas at room temperature, making administration and *in vivo* tracking challenging. To address these issues, Yao et al. designed a novel H_2_S donor, HSD-R, which combined cage carbonyl sulfide with ROS-responsive phenylboronic esters 164. When HSD-R hydrolyzed to form boronic acid in aqueous solution, H_2_O_2_ induced its oxidation to produce the fluorescent amino-compound HSD-RF and released COX. Commonly present CA further catalyzed the conversion of COX to H_2_S. After the donor released H_2_S, it can restore quenched red fluorescence, allowing quantitative analysis of H_2_S release kinetics and distribution *in vivo*. Researchers further elucidated the specific mechanisms of H_2_S protective effects, including reducing myocardial cell apoptosis and downregulating the expression of pro-apoptotic genes such as Bid, Apaf1, and the p53 signaling pathway. HSD-R not only exhibited excellent luminescent properties but also showed high selectivity for H_2_O_2_. Its development as a novel H_2_S donor with high conversion potential demonstrates promise in treating MI-related cardiac diseases.

After MI, excessive ROS induces cell apoptosis, exacerbating cardiac damage. Various ROS-responsive materials have shown promise in the treatment of MI-related cell apoptosis, with injectable hydrogels being particularly notable for their potential to improve heart function by inhibiting apoptosis and promoting angiogenesis. However, despite the encouraging progress made in both scientific research and clinical trials, the optimal injection parameters—including injection site, volume and speed—require further theoretical exploration and clinical validation. Additionally, for patients with varying degrees of MI or different ages, there is still a lack of scientific evidence and clear guidelines regarding the ideal properties of hydrogels, such as modulus, degradation rate and injectability.

### Ischemia-reperfusion injury

Ischemic myocardium often suffers further damage upon reperfusion, a phenomenon known as MIRI. After acute MI, thrombolytic therapy or PCI are the most effective measures, rapidly restoring myocardial perfusion, reducing infarct size, and improving prognosis. However, while these treatments restore blood flow to ischemic myocardium, they may also trigger reperfusion injury, diminishing effectiveness and exacerbating tissue damage 165. During reperfusion, the persistent opening of mitochondrial permeability transition pores (mPTP) and the intense production of ROS are the main damage mechanisms. The opening of mPTP leads to increased ROS production by mitochondria, further inducing myocardial cell apoptosis 166. Oxidative stress and mitochondrial dysfunction are key factors during myocardial ischemia and reperfusion. The treatment of MIRI is critically essential for the management of CVDs. It has the capacity to curtail the generation of ROS, reinstate the energy metabolism of cardiomyocytes, avert the demise of cardiomyocytes resulting from oxidative stress and energy disequilibrium, and safeguard cardiac function 167. Although there are many biomaterials designed for the mechanisms of MIRI, including exosomes, peptides and others, there is a lack of an effective system that ensures their targeted action at specific sites 168. The introduction of smart responsive systems, which can dynamically regulate the release of biomaterials in response to changes in the microenvironment, holds the potential to improve therapeutic outcomes. Given the critical role of ROS in MIRI and its association with mitochondrial dysfunction, ROS-responsive biomaterials could offer a promising therapeutic approach to mitigate oxidative stress, protect mitochondrial function, and reduce myocardial injury during reperfusion. Currently, the mechanisms of using biomaterials to treat MIRI mainly focus on regulating oxidative stress and restoring mitochondrial function. Through these mechanisms, it is possible to effectively alleviate MIRI, reducing cell apoptosis and tissue damage.

#### Regulation of mitochondria

Mitochondria play a crucial role in cellular oxidative phosphorylation. During ischemia and hypoxia, the intracellular partial pressure of oxygen decreases, leading to mitochondrial dysfunction in oxidative phosphorylation and damage to the electron transport chain. This damage reduces the activity of antioxidant enzymes such as superoxide dismutase (SOD), catalase, and glutathione peroxidase, thereby increasing intracellular ROS production during the reperfusion phase 169,170. Therefore, regulating mitochondrial function is crucial for treating MIRI. Current treatment strategies primarily aim to regulate related pathways and enhance mitochondrial autophagy to reduce ROS production in mitochondria, thereby alleviating MIRI.

Cyclosporin A (CsA) can inhibit the opening of mPTP, a channel considered crucial for reducing mitochondrial dysfunction and abnormal ROS production. Zhang et al. designed an innovative therapeutic approach by encapsulating SS-31 peptide-modified amphiphilic PLGA-thiosuccinyl-PEG (PLGA-TKPEG) in an injectable hydrogel, loading it with CsA nano-micelles (PTPSC) 171. This design aimed to treat MIRI by restoring mitochondrial function and reducing oxidative stress. PTPSC was cross-linked with the hydrogel through ROS-responsive borate ester bonds. After injection, this hydrogel responded to ROS in the cardiac microenvironment, releasing the loaded PTPSC. SS-31 peptide is a mitochondrial-targeting peptide that specifically concentrates on the mitochondrial membrane by interacting with cardiolipin expressed on the membrane, making its targeting independent of mitochondrial membrane potential, allowing effective targeting even during mitochondrial dysfunction. After injection into I/R-injured myocardium, this hydrogel effectively mitigated oxidative stress at the microenvironmental, cellular, and subcellular levels while inhibiting mitochondrial dysfunction.

Another study developed ROS-responsive nanoparticles, Poly(ethylene glycol)-b-Poly(propylene sulfide) (PEG-b-PPS), and used them to encapsulate and deliver ginsenoside Rg3 (PEG-b-PPS-Rg3) 172. FOXO3a is an important protein regulating antioxidant responses and enhances mitochondrial autophagy by activating the Sirt-1 pathway, reducing ROS production. Activated FOXO3a can mitigate oxidative stress damage in rat I/R models by enhancing SOD activity. Experiments indicated that Rg3 modulated FOXO3a and inhibited oxidative stress, inflammation, and fibrosis through downstream signaling pathways. Intramyocardial injection of PEG-b-PPS-Rg3 allowed Rg3 to be released in response to ROS at the injury site, significantly reducing mortality in I/R rats. This finding provides important evidence for the development of Rg3 as a therapeutic agent for CVDs and serves as a reference for the screening of natural product targets and exploration of their mechanisms.

Regulating mitochondrial function is essential for treating MIRI. Ischemia and oxidative stress lead to mitochondrial dysfunction and excessive ROS production, worsening cell damage. By modulating mitophagy, inhibiting mPTP opening, and restoring mitochondrial function, ROS production can be reduced, alleviating MIRI. ROS-responsive biomaterials, a promising new therapeutic approach, can mitigate oxidative stress and enhance mitochondrial function through targeted drug delivery to the damaged site. As these biomaterials evolve, they will enable more efficient and controllable treatments for MIRI by providing precise targeting and dynamic regulation. Combining nanotechnology with mitochondrial targeting offers potential for more accurate interventions at cellular and molecular levels, which can reduce side effects and improving the effectiveness and durability of therapy.

#### Antioxidant and anti-inflammatory

After MIRI, the production of ROS significantly increases. These ROS damage polysaccharides and oxidize proteins, leading to peroxidation of membrane fatty acids and the generation of bioactive substances such as prostaglandins, TXA2, and leukotrienes, thereby exacerbating I/R injury 173. The endogenous signaling molecule NO plays a critical role in preventing platelet aggregation, regulating smooth muscle cell phenotype, and maintaining endothelial cell function 174. However, in a pathological cardiovascular microenvironment, high levels of ROS inhibit the production of NO, undermining its protective effects 175. Therefore, scavenging excess ROS not only helps mitigate reperfusion injury but also maximizes the biological effects of NO. Similar to treatments for AS and MI, using ROS-responsive biomaterials for antioxidant and anti-inflammatory therapies is a key strategy for treating MIRI. Specific therapeutic mechanisms involve using antioxidants and anti-inflammatory drugs to modulate the ROS/NO balance, restoring a normal cardiovascular microenvironment.

Lee et al. reported a molecular-engineered solid polyoxalate copolymer (HPOX) nanoparticle, which exhibited stability and high specificity for H_2_O_2_ 38. The antioxidant and anti-inflammatory agent HBA were covalently linked to the main chain of HPOX, while an anti-apoptotic drug was loaded into HPOX for controlled release. In the presence of H_2_O_2_, the HPOX copolymer hydrolyzed, exerting its intrinsic antioxidant and anti-inflammatory effects while also releasing the anti-apoptotic drug to enhance therapeutic efficacy. Additionally, HPOX underwent chemiluminescent reactions with H_2_O_2_ in the presence of fluorescent compounds, enabling imaging of H_2_O_2_ generated during I/R injury. This nanoparticle can serve as a bioimaging agent and targeted DDS, delivering higher concentrations of drugs to affected areas during I/R.

**Figure 12:**
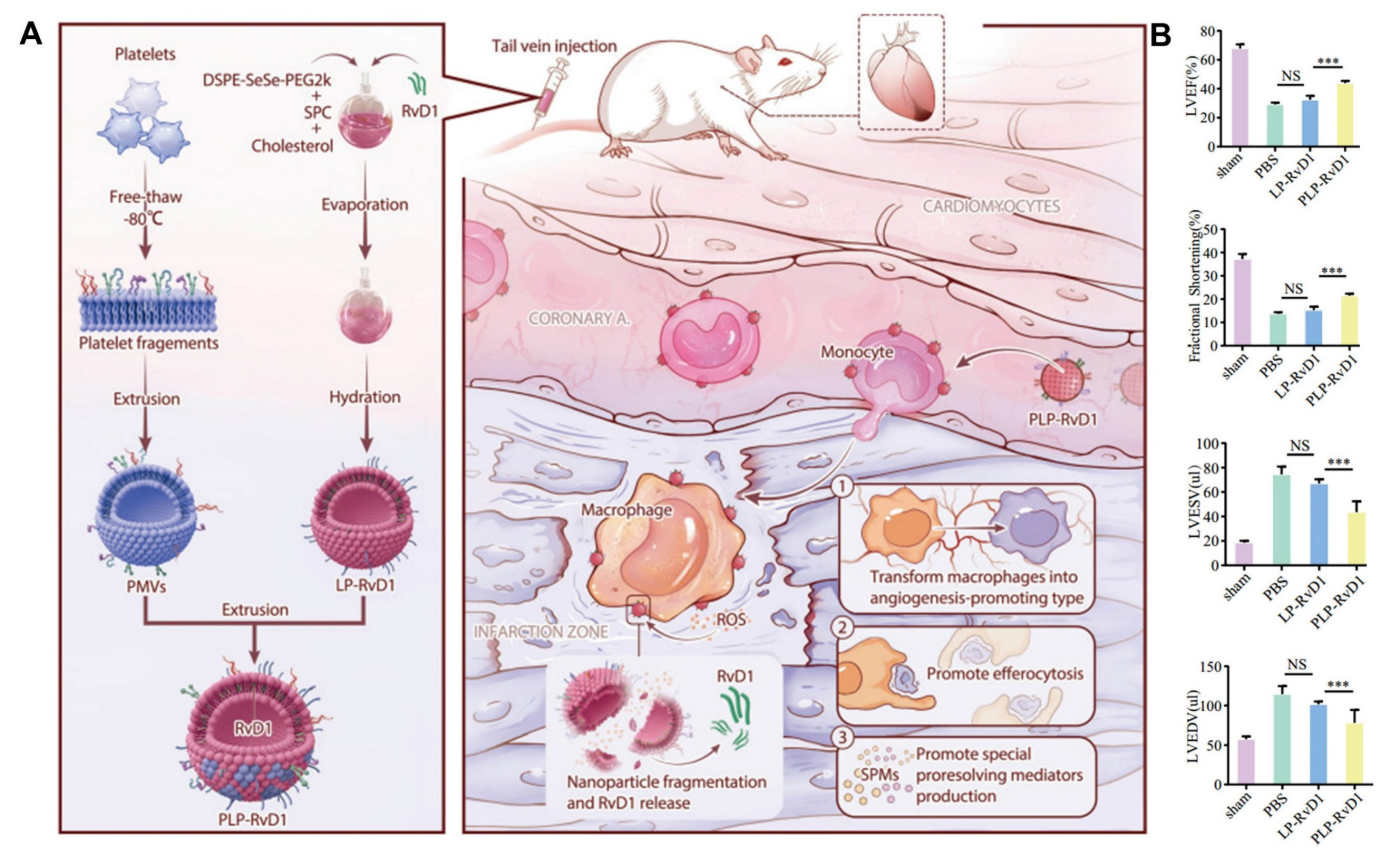
** (A)** Schematic of PLP-RvD1 fabrication and its targeting treatment for MIRI. **(B)** Cardiac protection efficiency of PLP-RvD1. A Cardiac function was assessed by echocardiography at 4 weeks after treatment. Adapted with permission from 26, an open access article published 2022 by Springer Nature under a CC-BY4.0 license.

Resolvin D1 (RvD1) is a specialized pro-resolving mediator (SPM) recognized for its crucial role in guiding the active resolution of acute inflammation and promoting macrophage clearance of apoptotic and necrotic cells. Inflammation resolution is a key process for cardiac function recovery after MIRI. Weng et al. designed a platelet-mimicking, ROS-responsive RvD1 delivery platform (PLP-RvD1), formed by ROS-responsive liposomes loaded with RvD1 and a PM **(Figure 12A)** 26. The PM can chemotactically guide monocytes to the site of cardiac injury and enable local release of RvD1 through ROS-responsive diselenide bonds in the delivery platform. In *in vivo* applications, PLP-RvD1 effectively improved ventricular remodeling in MI/R-induced mice and significantly protected cardiac function. To assess the therapeutic efficacy of PLP-RvD1, cardiac function in mice subjected to MI/R injury was evaluated via echocardiography following 4 weeks of treatment with PBS, LP-RvD1, or PLP-RvD1. As depicted in **Figure 12B**, the PLP-RvD1-treated group demonstrated the highest preservation of left ventricular ejection fraction (LVEF) compared to the other groups. Additionally, other echocardiographic indicators, such as left ventricular end-systolic volume (LVESV) and left ventricular end-diastolic volume (LVEDV), exhibited consistent trends. This delivery platform integrates drug biostability, targeted delivery, and controlled release, demonstrating substantial clinical translation potential.

In addition to exogenous antioxidant therapy, endogenous carbon monoxide (CO) has been widely reported to possess anti-inflammatory and anti-apoptotic effects. After IR, excessive production of O_2_⁻ occurs, which reacts with NO to form excessive peroxynitrite (ONOO⁻). This leads to damage of the calcium pump, apoptotic cell death, and triggers an inflammatory cascade. Zhang et al. investigated a novel CO donor, consisting of macrophage membrane-coated PLGA nanoparticles and PCOD585 (M/PCOD@PLGA) 176. PCOD585, as a CO donor responsive to ONOO⁻, reacted with ONOO⁻ and simultaneously released CO. Further *in vivo* studies in mice demonstrated that M/PCOD@PLGA improved cardiac function after MIRI by inhibiting excessive inflammation and reducing cell apoptosis.

**Figure 13:**
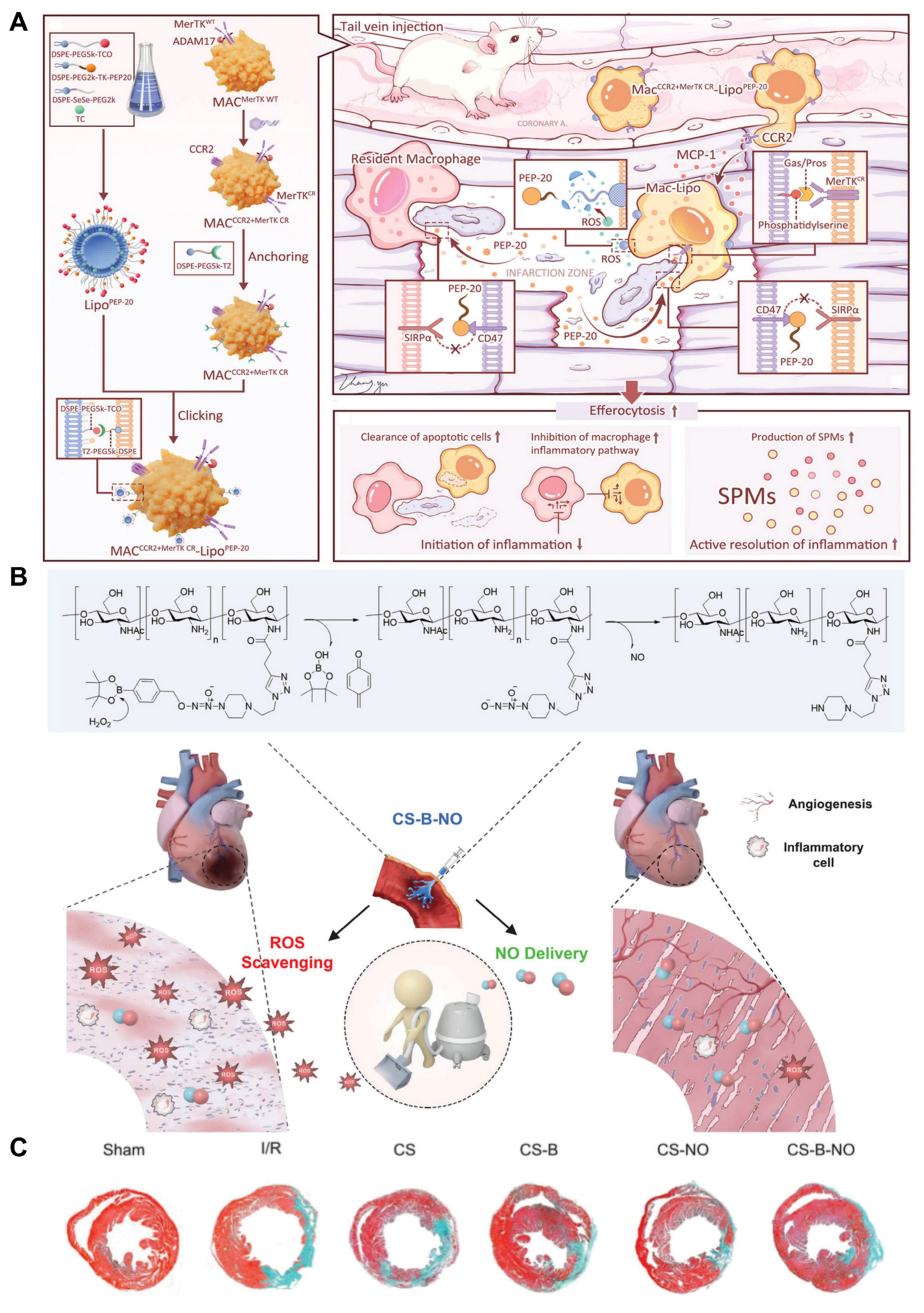
**(A)** Schematic illustration of MAC^CCR2+MerTK CR^-Lipo^PEP-20^ as a potential candidate to reconstruct efferocytosis post-MI/R injury. Adapted with permission from 177, an open access article published 2024 by Wiley-VCH GmbH under a CC-BY4.0 license.** (B)** Schematic illustration of the treatment of I/R heart injury by CS-B-NO. **(C)** Masson's trichrome was performed, and infarcted size was quantified accordingly. Adapted with permission from 178, an open access article published 2022 by Wiley-VCH GmbH under a CC-BY4.0 license.

Efferocytosis not only prevents the spread of inflammation by clearing apoptotic cells but also actively initiates inflammation resolution by promoting the production of specialized SPMs. However, the upregulation of the “don't eat me” signal CD47 in MI/R-induced apoptotic cells inhibits efferocytosis. Tan et al. developed a synergistic drug delivery strategy based on transgenic macrophages, where exogenous macrophages were able to effectively counteract CD47 inhibition of efferocytosis through ROS-responsive liposomes loaded with PEP-20 (a CD47 antagonist) **(Figure 13A)** 177. The liposomes were connected to a hydrophobic tail via thio-ketone bonds. In the presence of pathological concentrations of H_2_O_2_, PEP-20 could undergo responsive cleavage from the tail, thereby improving macrophage efferocytosis after MI/R, inhibiting inflammation initiation, and promoting resolution. This strategy provides a new perspective for immunomodulatory treatment of MI/R. Numerous studies indicate that NO is a multifunctional signaling molecule that plays a crucial role in angiogenesis and cardiac protection. Inhaling NO can inhibit leukocyte adhesion and activation on damaged endothelium, thereby reducing excessive ROS production in the heart. Consequently, researchers have developed various NO-containing biomaterials for targeted delivery and controlled release of NO. Hao et al. designed a novel hydrogel, CS-B-NO, which had dual functions of ROS scavenging and NO release **(Figure 13B)** 178. In an environment where ROS was present, the borate ester groups were activated, leading to NO release. In a mouse model of I/R injury, researchers injected the CS-B-NO hydrogel *in situ* into the ischemic myocardium. In the early stages, the CS-B-NO hydrogel reduced myocardial cell apoptosis and alleviated the inflammatory response. In late stage, CS-B-NO hydrogel alleviated cardiac remodeling by reducing infarct size** (Figure 13C)**. Additionally, researchers explored the cardioprotective mechanisms of CS-B-NO: regulating the ROS/NO balance can activate antioxidant defense systems. On one hand, the CS-B-NO hydrogel activates the Nrf2 signaling pathway by enhancing Keap1 S-nitrosylation, protecting the heart from oxidative stress. On the other hand, this hydrogel inhibits the phosphorylation of the upstream IKK/IkBα-mediated NF-kB signaling pathway, reducing NF-kB activation and the expression of downstream pro-inflammatory factors.

### Vascular restenosis

Restenosis refers to the re-narrowing of a blood vessel after interventional procedures such as angioplasty or stent implantation, leading to obstructed blood flow 179. For instance, restenosis of the coronary arteries diminishes the blood supply to the myocardium, thereby triggering angina 180. Restenosis of the cerebral arteries may result in inadequate cerebral blood supply, giving rise to symptoms like dizziness and IS. Treating vascular restenosis is of utmost importance for the management of CVDs. This measure can not only guarantee the blood supply to vital organs such as the heart and brain, but also reduce the risk of thrombosis, enhance the effectiveness of interventional treatments, and improve the prognosis of patients. The primary cause of restenosis is the formation of neointima on the vessel wall, a process known as intimal hyperplasia. Intimal hyperplasia is characterized by excessive proliferation and phenotypic changes of VSMCs. During this process, the synthesis and secretion of ECM components and cytokines (such as platelet-derived growth factor (PDGF)) promote VSMC proliferation and migration.

Mechanical injury and inflammatory stimuli lead to the transformation of VSMCs from a contractile to a secretory phenotype, becoming the main component of intimal hyperplasia. Research indicates that inflammation plays a critical role in the process of restenosis. The intimal surface contains numerous inflammatory cells that produce harmful ROS and secrete enzymes that degrade ECM components, facilitating fibroblast migration 181. ROS are generated during inflammation and mechanical injury, participating in the activation of various signaling pathways, further promoting the proliferation, migration, and phenotypic transformation of VSMCs. ROS can also activate redox-sensitive transcription factors, such as NF-κB and AP-1, which further enhance the inflammatory response and promote the progression of intimal hyperplasia. Current clinical treatment methods include drug-eluting stents (DES) and balloon angioplasty. DES release drugs locally to inhibit smooth muscle cell proliferation, thereby reducing the occurrence of restenosis. However, post-stent implantation, issues such as delayed endothelial repair and mechanical damage to the blood vessel caused by the stent material remain problematic. Given the critical role of ROS in the progression of restenosis, ROS-responsive biomaterials that target inflammatory pathways and VSMC behavior may offer a promising strategy for mitigating restenosis following interventional procedures. The vascular endothelium is the inner layer of the vessel wall, serving functions such as anticoagulation, anti-inflammation, and regulation of vascular tone. The repair and regeneration of damaged endothelial cells are crucial for maintaining normal vascular function. An intact and functional endothelial layer can inhibit excessive VSMC proliferation, prevent platelet aggregation, and reduce inflammatory responses, thereby decreasing the incidence of restenosis 182. The NO released by healthy endothelial cells inhibits VSMC activity and vascular constriction. Current treatment strategies include targeted delivery of anti-restenosis, antioxidant, and anti-inflammatory drugs, as well as the introduction of components that promote endothelial proliferation for gene therapy aimed at endothelial regeneration.

**Figure 14:**
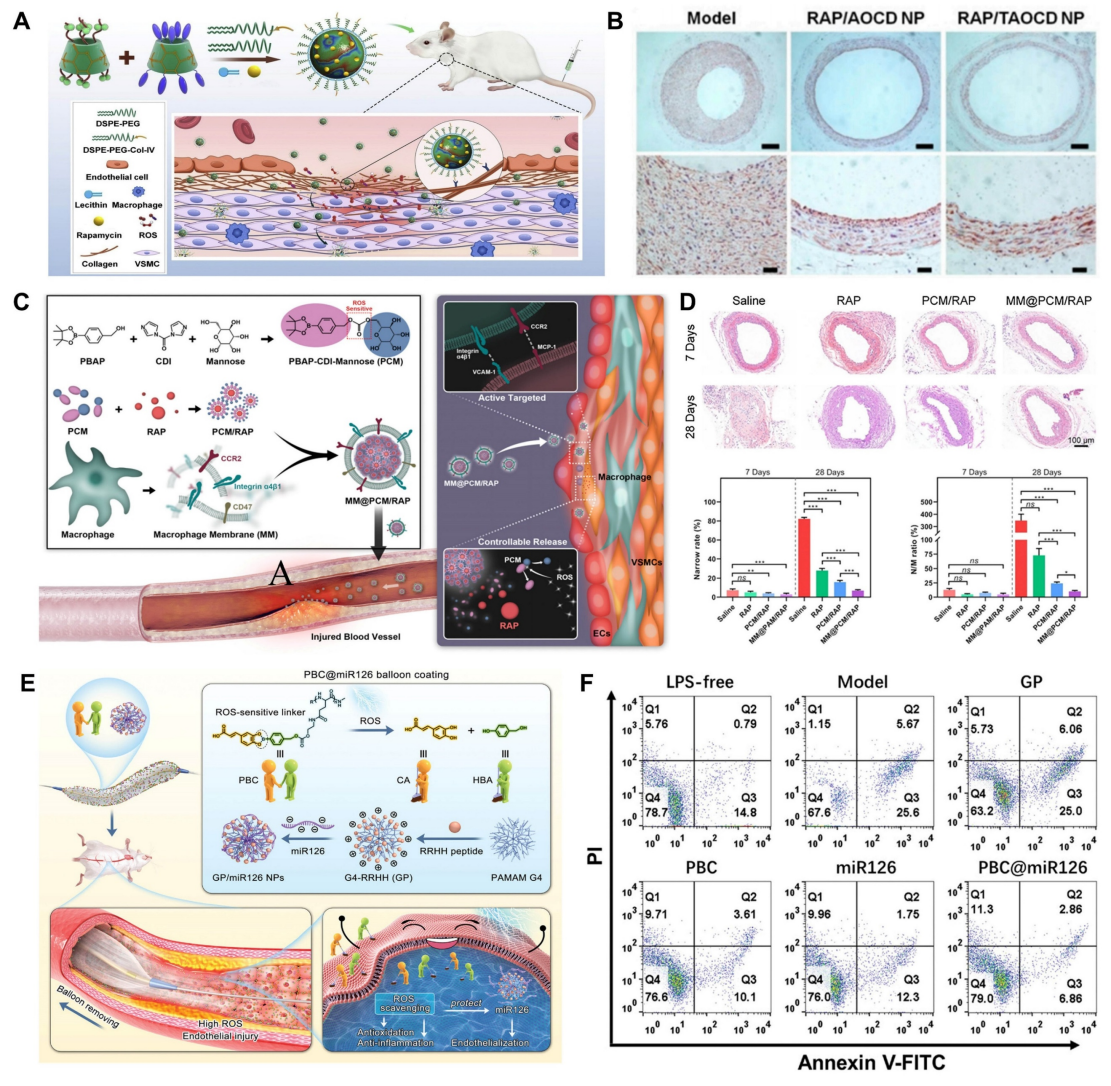
**(A)** Design and engineering of pH/ROS dual-responsive nanotherapies for targeted treatment of restenosis. **(B)** Immunohistochemical analysis of arterial cross-sections from a rat carotid restenosis model treated with non-targeting or targeting dual-responsive nanotherapies, stained with α-SMA antibodies. Adapted with permission from 184, an open access article published 2019 by Elsevier Ltd under a CC-BY4.0 license. **(C)** Illustrations of MM@PCM/RAP for the treatment of IH. **(D)** H&E staining of carotid artery sections from mouse model after different treatments for 7 and 28 days. Narrow rate and N/M ratio of carotid artery sections (n=6, mean±SD). Adapted with permission from 185, copyright 2021, Springer Nature. **(E)** Schematic illustration of vascular restenosis prevention by ROS-responsive/scavenging PBC@miR126-coated balloon. **(F)** Flow cytometric profiles of apoptosis in different groups. Adapted with permission from 186, copyright 2023, Wiley-VCH GmbH.

Considering that the inflammatory microenvironment typically exhibits weak acidity and high ROS levels, Feng et al. developed a ROS-responsive nano-therapy 183. They synthesized a ROS-responsive material (Ox-bCD) using β-CD as the parent compound through simple chemical modifications, and utilized RAP as the delivery drug. In DES, RAP reduces restenosis by inhibiting VSMC migration and proliferation. The migration and proliferation of VSMCs are closely linked to the pathogenesis of restenosis. As an mTOR inhibitor, RAP exerts its pharmacological activity in the cytoplasm by binding to FKBP12. After intravenous injection, these responsive NPs effectively targeted the injured vessels through passive targeting. Upon responding to ROS in the inflammatory microenvironment, the phenylboronate group hydrolyzed Ox-bCD and released RAP. After effective internalization by rat VSMCs, the anti-proliferative and anti-migratory activities of RAP were significantly enhanced. This design effectively validates a nano-therapy triggered by the inflammatory microenvironment that preferentially releases drugs at the disease site, serving as an effective targeted treatment for restenosis. Similar to the therapy developed by Feng et al. Zhang et al. designed a dual-responsive nano-therapy system based on β-CD, called RAP/AOCD NPs, which combines pH and ROS-responsive materials to encapsulate the candidate drug RAP **(Figure 14A)** 184. In low pH or high H_2_O_2_ environments, the loaded RAP molecules can be triggered to release. Compared to free drugs and non-responsive or single-responsive nano-therapies, RAP/AOCD NPs exhibited stronger anti-migratory and anti-proliferative activities in VSMCs. To achieve more efficient targeting, the research team modified the surface of AOCD NPs with a peptide sequence (KLWVLPKGGGC) that targets type IV collagen. The* in vivo* efficacy of targeted versus non-targeted nanotherapy was compared in a rat model of restenosis. Immunohistochemical analysis indicated that, compared to RAP/AOCD NPs, RAP/TAOCD NPs more effectively inhibited the proliferation of VSMCs in the intimal region after treatment. This suggests that partial targeting modification can further enhance the *in vivo* anti-restenosis efficacy of nanotherapies **(Figure 14B)**. To enhance drug targeting at inflammatory lesions, Liu et al., inspired by previous studies, applied a biomimetic cell membrane camouflage strategy to a nano DDS for treating restenosis **(Figure 14C)** 185. They designed an amphiphilic carrier based on macrophage membrane wrapping (MM@PCM/RAP) for delivering the drug RAP. ROS-responsive amphiphilic molecule PBAP-CDI-Mannose (PCM), which was synthesized by conjugating 4-(hydroxymethyl) phenylboronic acid pinacol ester (PBAP) with *D*-mannose, could dissolve and load RAP, triggering localized drug release in the presence of high concentrations of ROS in pathological environments. The inherent “homing” ability of MM enabled this delivery system to selectively accumulate at damaged vascular lesions, enhancing localized drug release. This biomimetic strategy not only enhances the bioavailability of RAP but also effectively reduces systemic side effects. In an animal experiment using a carotid artery injury mouse model, different formulations were injected. After treatment, H&E staining was performed to validate tissue morphology. The saline group showed significant IH at both 7 and 28 days, while all three RAP treatment groups exhibited marked inhibition, with the MM@PCM/RAP group demonstrating the strongest anti-restenosis effect **(Figure 14D)**. This biomimetic nano DDS demonstrates significant clinical application potential and is expected to become an important tool for future vascular disease treatment.

Excessive inflammation and oxidative stress can lead to tissue and cell damage, impairing endothelial function and exacerbating intimal hyperplasia and restenosis. Excessive production of ROS is considered a primary driving factor in this process. To address this issue, Zhao et al. developed a novel therapeutic strategy that incorporated ROS-responsive/scavenging prodrugs into balloons for treating restenosis** (Figure 14E)** 186. They designed a reversible phenylboronate caffeic acid (CA) prodrug (PBC) specifically for local high ROS levels, enabling controlled dual drug release on demand. Under high ROS levels, PBC released CA and p-HBA, both of which exhibited effective antioxidant and anti-inflammatory effects by scavenging ROS, thereby modulating the vascular microenvironment and protecting endothelial function. Additionally, to accelerate endothelial regeneration, the research team introduced pro-endothelial microRNA-126 for gene therapy. Cell experiments assessed the protective effect of coatings on vascular cell survival. Flow cytometric profiles showed that the combination of PBC and miR126 achieved the best anti-apoptotic effect **(Figure 14F)**. This integrated ROS-responsive/scavenging prodrug/gene delivery platform provides new insights for improving the long-term outcomes of balloon therapy and offers broad application prospects for next-generation drug-eluting balloons and other cardiovascular treatments. In the field of anti-restenosis therapy, various drug nanocarriers have been extensively studied and applied, including inorganic NPs, polymer NPs, and liposomal NPs. These nanocarriers enhance drug targeting, stability, and bioavailability through different mechanisms. In recent years, cell membrane-coated biomaterials have gained increasing attention as an emerging concept in precision medicine. By combining the biomimetic features of cell membranes with the multifunctionality of nanoparticle cores, it significantly enhances drug delivery efficiency. Zhao et al. designed a multimodal nanocluster formed by the self-assembly of monomeric nanoparticles, with a surface coated in PM 187. This design leveraged the lesion-targeting capabilities and biocompatibility of the PM coating. It also utilized the multifunctionality of the nanocluster to achieve precise delivery of anti-restenosis drugs. The structural design of the nanocluster allowed it to respond to ROS at the target site via a “cluster bomb” chemical mechanism, transitioning from large to small size. This feature not only enhances the retention time of the drug at the lesion site but also improves its permeability within tissues. Researchers further loaded an emerging anti-restenosis drug, RVX-208 (a small-molecule inhibitor), into these nanoclusters. RVX-208 effectively intervened in the proliferation and migration of VSMC by selectively blocking the intervention target for restenosis (BET), thereby reducing intimal hyperplasia and the occurrence of restenosis. In a rat balloon angioplasty model, a single intravenous injection of RVX-208-loaded nanoclusters (at a dose of 10 mg/kg) significantly alleviated restenosis, demonstrating its strong therapeutic potential. This study shows that this multimodal nanocluster not only possesses excellent targeting and tissue permeability but also has the ability to release drugs in response to ROS. Its unique design and superior performance make it a promising platform for developing next-generation intravascular therapies, potentially leading to revolutionary breakthroughs in anti-restenosis treatment.

The generation of ROS plays a critical role in the process of restenosis. ROS activate various signaling pathways, further enhancing the inflammatory response and promoting the proliferation and migration of VSMCs, thereby exacerbating intimal hyperplasia. The application of ROS-responsive biomaterials offers a novel therapeutic strategy by responding to oxidative stress in the damaged area and precisely release drugs, such as those inhibiting VSMC proliferation (e.g., RAP), effectively slowing restenosis. Furthermore, the use of gene therapy to directly target key regulatory genes involved in VSMC proliferation and migration, or the application of CRISPR-based technologies to modulate inflammatory pathways, represents an innovative direction for improving restenosis treatment.

#### Others

##### Peripheral artery disease

PAD is a common form of AS that primarily affects the peripheral arteries, particularly those in the lower limbs 188. Endothelial dysfunction and the accumulation of LDL in the arterial walls contribute to the gradual narrowing and blockage of these arteries, which negatively impacts blood supply to surrounding tissues. The primary goal of PAD treatment is to restore blood flow to the distal limbs using vascular reconstruction techniques, such as bypass surgery and angioplasty 189. However, some patients may not be suitable candidates for surgical intervention due to anatomical locations or overall health conditions. Research indicates that patients with PAD have elevated levels of oxidative stress and inflammation in their arterial walls. Excessive ROS disrupt the homeostasis of vascular tone, leading to arterial vascular lesions. As a result, there is growing interest in utilizing ROS-responsive biomaterials to deliver antioxidants and anti-inflammatory agents to prevent the excessive accumulation of ROS in endothelial cells during PAD treatment.

Salvianolic Acid B (SAB) is a natural phenolic compound extracted from the root of Salvia miltiorrhiza, known for its significant antioxidant properties that protect vascular endothelial cells from oxidative damage. Chen et al. developed a shear-thinning laponite hydrogel containing ROS-responsive dextran-based nanoparticles for loading SAB 41. This hydrogel served as a drug carrier that could hydrolyze in an ROS environment, transforming into hydrophilic dextran and facilitating controlled drug release. *In vitro* experiments showed that SAB scavenged ROS and protected human umbilical vein endothelial cells from oxidative damage by reducing inflammation and apoptosis. Cytochrome P450 epoxygenase 2J2 (CYP2J2) and its catalytic product, epoxyeicosatrienoic acids, possessed multiple biological activities, including pro-angiogenic, anti-inflammatory, and cardiovascular protective effects. These effects are beneficial for reversing ischemia and restoring local blood flow in critical limb ischemia. Gui et al. designed a nanoparticle-based delivery system for pcDNA3.1-CYP2J2 plasmid DNA (pDNA), composed of a novel three-armed star-shaped block copolymer (3S-PLGA-po-PEG) 190. The polymer contained peracetic acid (po), which could respond to H_2_O_2_ to release the loaded pDNA. Compared to the control group treated with naked pDNA, muscle injection of this nanoparticle complex significantly accelerated blood flow recovery in the hind limbs of ischemic mice and promoted muscle repair.

##### Vascular endothelial cell dysfunction

Endothelial cells (ECs) are the primary component of the vascular inner wall and play a critical role in maintaining vascular homeostasis and regulating vascular function. Damage and dysfunction of ECs manifest as impaired endothelial-dependent vasodilation, increased oxidative stress, chronic inflammation, leukocyte adhesion, heightened permeability, and cellular senescence 191. These dysfunctions are early key events in the development of CVDs 192. In CVDs, the excessive production of ROS induces endothelial cell dysfunction by damaging the intracellular environment, impairing NO production in the endothelium, and increasing vascular permeability, ultimately leading to the progression of CVDs. Utilizing ROS-responsive biomaterials to maintain and restore the integrity of endothelial cells is crucial for normal vascular function and the recovery from related diseases.

Qin et al. developed a biomimetic ROS-responsive nano-therapy agent disguised with red blood cells (RBC-LVTNPs) for repairing EC damage 193. Under ROS stimulation, the prodrug lovastatin (LVT) could be precisely released at the site of EC lesions, improving EC function, enhancing NO production, and suppressing inflammatory factors. Additionally, the low oscillatory shear force at the EC site promoted the uptake of RBCM-derived vesicles by ECs, further enhancing targeting to the lesion. This nano-therapy agent offers a promising strategy for developing novel nanotherapeutics using biomimetic cell membrane technology, potentially playing an important role in the treatment of CVDs.

##### Abdominal aortic aneurysms

Abdominal aortic aneurysm (AAA) is a highly lethal aortic disease, typically suspected when the diameter of the aortic wall exceeds 50% of the normal abdominal aorta diameter or exceeds 30 mm, and it often leads to acute vascular rupture. VSMCs are the main intrinsic cells in the arterial wall and play a key role in synthesizing the extracellular matrix to maintain the structure, vascular tone, and blood flow of the aorta. Under pathological conditions, VSMCs proliferate excessively and migrate from the media to the intima, resulting in vascular occlusion. Currently, mechanical intervention is the only effective treatment to prevent aneurysm-related mortality. However, open surgery is associated with perioperative mortality and morbidity. Therefore, the use of effective drugs to reduce AAA expansion and prevent AAA rupture is necessary. ROS have been shown to play a promotive role in AAA, leading to the apoptosis of intimal VSMCs.

Cheng et al. developed a ROS-responsive nanomaterial (CROR NP) targeted at AAA, which effectively delivered the therapeutic molecule RAP to the affected site and successfully inhibited the expansion of AAA in rats 194. In* in vivo* experiments, compared to PR NP, which loaded RAP but lacked ROS responsiveness, OR NP could reduce ROS production and inhibit calcification by downregulating the expression of MMPs and pro-inflammatory cytokines/chemokines. Notably, this study used ligand- and macrophage membrane-mediated synergistic targeting, enhancing targeted drug delivery via receptor-mediated endocytosis. This study provides a potential therapeutic strategy for clinical AAA treatment by improving the inflammatory microenvironment of aneurysms, protecting VSMCs, and inhibiting the progression and rupture risk of AAA.

ROS-responsive biomaterials are commonly used as drug carriers for the treatment of CVDs, but their effectiveness may be limited by the pharmacological actions of the drugs themselves.

A promising new approach is the design of materials with intrinsic biological activity. Lin et al. designed a nano-micelle TPTN assembled from PBE and TP complexes 195. In the presence of ROS, the hydrophilic molecules formed by the cleavage of 4-Hydroxyphenylboronic acid pinacol ester in the nano-micelles effectively scavenged ROS, inhibited the migration and activation of inflammatory cells, and protected VSMCs from oxidative stress, calcification, and apoptosis. Notably, when TPTN was used as a targeted drug delivery nanocarrier, it could specifically deliver and trigger the release of anti-aneurysm drugs at the site of abdominal aortic injury. This design, as a multi-bioactive nanotherapy and ROS-responsive drug delivery carrier, provides new insights for the treatment of AAA and other CVDs.

## Conclusion and perspective

This review systematically summarizes the application of ROS-responsive biomaterials in CVDs, encompassing their roles in early diagnosis and treatment, with a particular focus on therapeutic strategies using ROS-responsive biomaterials to address the pathological role of ROS in various CVDs. Excessive ROS is implicated in the pathological processes of CVDs, making it a potential stimulus marker to trigger *in situ* assembly or disassembly of biomaterials within the cardiovascular pathological microenvironment, or a target to guide biomaterial enrichment at the lesion site. These ROS-responsive biomaterials not only address limitations in traditional CVDs diagnosis and treatment but also offer more effective therapeutic approaches.

**Figure 15:**
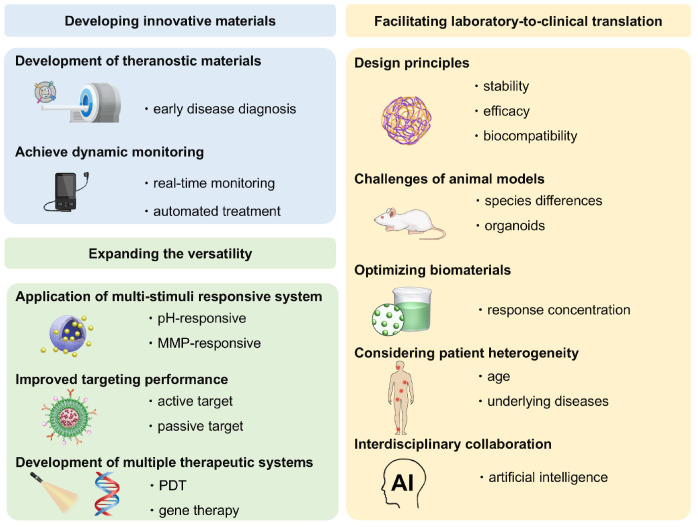
The perspective of ROS-responsive materials in CVDs.

While ROS-responsive biomaterials hold great potential for personalized CVDs management, these systems are still in their developmental stages and require careful consideration of their application scope, accounting for the unique pathophysiological characteristics of different CVDs and individual patient variability **(Figure 15)**. Several challenges remain to be addressed by researchers:

### 1. Developing innovative materials

(1)** Development of theranostic materials:** Theranostic materials, capable of real-time monitoring of CVDs progression, are crucial for personalized medicine, enabling flexible adjustment and optimization of treatment plans. In particular, the unique microenvironment of the cardiovascular system provides an opportunity for the development of responsive probes and vectors, enabling site-specific diagnostic imaging and drug release. For example, integrating ROS-responsive biomaterials with magnetic nanoparticles (MNPs) can facilitate diagnostic and bioimaging functionalities. MNPs coupled with monocyte chemoattractant protein-1 peptide can detect the progression of atherosclerotic plaques, enabling imaging. Simultaneously, ROS-responsive biomaterials can serve as DDS, combining therapeutic and imaging capabilities, revealing the spatial and temporal distribution of drugs, promoting pharmacokinetic studies, and facilitating early disease diagnosis.

(2) **Achievement of dynamic monitoring:** ROS-responsive materials that integrate diagnostic and therapeutic functions could enable patients to benefit from a simpler treatment regimen. The development of wearable ROS-responsive sensors is an emerging area of research that holds great promise. These sensors are designed to perform continuous, real-time monitoring of ROS levels* in vivo*, providing an ongoing stream of data that reflects the oxidative stress status of the body. By combining this sensor technology with an autonomous therapeutic system, it becomes possible to create a closed-loop feedback system where, upon detecting elevated ROS levels that signal a potential CVDs event, the therapeutic system automatically releases the appropriate drug. This seamless integration of real-time monitoring and automated treatment not only simplifies the management of CVDs but also empowers patients to have more control over their health. Additionally, this system allows healthcare providers to remotely monitor treatment effects, assess the efficacy of the therapy by observing changes in ROS levels, and intervene proactively if necessary, marking a shift from reactive to proactive, continuous care.

### 2. Expanding the versatility of ROS-responsive materials

(1)** Application of multi-stimuli responsive systems:** Many neurodegenerative diseases and cancers, similar to CVDs, share the common features of high ROS and oxidative stress. In complex cases, a single ROS stimulus may not meet therapeutic needs, potentially leading to drug release in non-target areas, reducing therapeutic efficacy and potentially causing side effects. Moreover, the pathological processes of CVDs are complex and diverse, involving other pathological features besides ROS, such as acidic environments and inflammatory responses. These factors collectively contribute to disease progression, making single ROS-responsive materials insufficient for comprehensive and effective treatment. Not only in treatment but also in diagnosis, similar issues exist. In disease diagnosis, a single target may sometimes fail to provide sufficient information, leading to false-positive signals. This requires further optimization of the responsive components of the biomaterials to align more closely with the levels of ROS and other biomarkers, such as inflammatory mediators, hypoxia, or altered pH, within the cardiovascular microenvironment. Therefore, designing multi-stimuli responsive systems, such as dual or multi-responsive materials combining ROS with low pH or other biomarkers, can enhance the accuracy and effectiveness of diagnosis and therapy. Future efforts should also focus on combining advanced imaging techniques and molecular biology tools to monitor and quantify the changes in various biomarkers. This approach will not only improve the targeting and precision of drug release but also enable the reverse engineering of biomaterials, allowing for their adaptation to specific pathological features.

(2)** Improved targeting performance:** Targeted approaches can effectively deliver ROS-responsive biomaterials to the desired organs. By actively targeting overexpressed proteins, antibodies, or specific peptides on disease-associated receptors, therapeutic agents can be delivered to specific cells, increasing their accumulation at the site of injury. This strategy is known as active targeting. ROS-responsive materials can be functionalized with ligands that specifically recognize disease-associated receptors in CVDs, enabling precise and controlled drug release in response to oxidative stress within the diseased microenvironment. For passive targeting, pathological processes such as altered vascular permeability, endothelial cell dysfunction, inflammation, and vascular remodeling, commonly associated with CVDs, serve as potential targets for DDS. ROS-responsive materials can reach the tissues of CVDs through the enhanced permeation and retention effect. Owing to their sensitivity to oxidative stress, these materials undergo alterations in solubility or structural conformation upon exposure to elevated ROS levels, thereby enabling localized drug release while minimizing systemic side effects. Additionally, another approach for targeted delivery of ROS-responsive biomaterials in CVDs involves preparing biomimetic nanoparticles using cell membranes with homing characteristics, which are selectively phagocytosed by immune cells and then transported to the target site via cellular carriers.

(3) **Development of multiple therapeutic systems:** In future studies, researchers can look at the development of multi-therapeutic systems. For example, PDT based on ROS-responsive biomaterials can be used to treat CVDs. By stimulating photosensitizers with specific wavelengths of light to produce singlet oxygen, the mTOR pathway can be modulated to promote cholesterol efflux from foam cells. Another promising approach is to combine ROS-responsive materials with stem cell therapy. ROS-responsive hydrogels or nanoparticles can serve as carriers for stem or progenitor cells, providing a controlled microenvironment that promotes cell survival, differentiation and integration into damaged tissues. By using ROS-responsive materials, the release of growth factors or signaling molecules can be precisely controlled to accelerate tissue regeneration and enhance repair of ischemic or infarcted cardiac tissue. In addition, ROS-responsive biomaterials can be incorporated into gene therapy strategies to deliver therapeutic nucleic acids to target cells to alleviate the pathological process of CVDs caused by abnormal gene expression. ROS-responsive delivery systems offer a promising strategy to prolong nucleic acid metabolism and improve endocytosis.

### 3. Facilitating laboratory-to-clinical translation

(1)** Design principles:** In the field of CVDs treatment, focusing on the design of nanoparticles and hydrogels is essential for precision therapy, optimizing efficacy and ensuring patient prognosis. ROS-responsive biomaterials must overcome challenges in stability, efficacy, and biocompatibility for successful clinical translation. Several key principles must be followed during the development process. Primarily, the biostability and biocompatibility of these novel materials must be prioritized to ensure that their *in vivo* degradation products are non-toxic. The mechanical environment of the cardiovascular system is complex because of the fluctuating stimuli from the beating heart and blood flow. When nanoparticles are used to deliver drugs, they must withstand shear forces from the blood flow, requiring cross-linking to strengthen their core and ensure drug-loaded particles remain stable, preventing premature drug leakage. Hydrogels used for myocardial repair must mimic the elasticity of cardiac tissue and adapt in real-time to the mechanical properties of the heart to avoid being too stiff or too soft, which could affect myocardial function. Enhancing the targeting or multifunctionality of ROS-responsive biomaterials by attaching different functional groups may affect the stability of conjugated biomolecules, altering the overall chemical structure and biological effects of the material. Therefore, special attention must be paid to these aspects during both material design and clinical translation. Furthermore, the clinical translation of biomaterials is often hindered by the complexity of their structure and mechanisms. To facilitate the clinical application of ROS-responsive biomaterials, the design should emphasize structural simplification, ease of manufacturing, and safety, bridging the gap between experimental research and clinical practice. Future research should focus on refining the chemical structure and physical properties of these materials to ensure robust* in vivo* biocompatibility and precise drug release profiles, thus laying a solid foundation for their eventual clinical application.

(2) **Challenges of animal models:** Currently, research on ROS-responsive biomaterials primarily focuses on preclinical stages. While ROS-responsive biomaterials demonstrate promising effects *in vitro* and in animal models, these findings may not directly translate to human clinical trials due to the inherent limitations of animal models in fully replicating the complex pathological characteristics and treatment responses of human CVDs. In the study of the cholesterol synthesis inhibitor mevastatin, differences in the hepatic metabolism of lipoproteins across species result in varying effects. Mevastatin has little effect on traditional rodent models, but shows significant efficacy in canine and non-human primate models. In such situations, researchers can use different animal models to conduct experiments, reducing the impact of species-specific differences, ensuring the broad applicability of the study results, and better predicting the potential therapeutic effects and side effects of biomaterials in humans. In recent years, researchers have begun using human-derived organoids, such as those derived from induced pluripotent stem cells or other sources, to replicate specific aspects of the* in vivo* heart and vasculature in order to model various CVDs 197. Cardiovascular organoids complement traditional animal models, providing valuable tools for preclinical research in the diagnosis and treatment of CVDs.

(3) **Optimizing biomaterials:** Despite the promising potential of ROS-responsive biomaterials in DDS, their clinical translation for CVDs faces challenges. These arise from dynamic fluctuations in ROS levels within the complex pathological microenvironment. CVDs progress through multiple stages, each with distinct pathological features. ROS concentrations and species vary across these stages. Therefore, understanding ROS profiles—such as their spatiotemporal distribution, concentration thresholds, and oxidative stress patterns—is essential for optimizing ROS-responsive biomaterials. Firstly, selecting the appropriate ROS-responsive bonds is crucial. The chemical responsiveness of these bonds must align with disease-stage-specific ROS levels. Secondly, biomaterial properties like degradation kinetics and targeting ligands should be designed to adapt to the evolving microenvironment of specific CVDs. For instance, incorporating inflammation-responsive motifs can target acute MI, while shear stress-sensitive modules are useful for atherosclerotic plaques. By synchronizing material responsiveness with stage-specific ROS dynamics and microenvironmental factors, precision therapeutic strategies can be developed. This will enable better control of drug release and improve treatment efficacy in CVDs.

(4) **Considering patient heterogeneity:** Patient heterogeneity, such as age, gender, underlying comorbidities (e.g. diabetes), and lifestyle factors (e.g. smoking, diet), plays a significant role in the oxidative stress levels present in individuals with CVDs. For instance, older patients may exhibit a different ROS profile compared to younger patients, due to age-related changes in the immune system and oxidative metabolism. Older individuals often have a lower antioxidant capacity, leading to elevated ROS levels in response to injury or disease, which may alter the release profile and efficacy of ROS-responsive biomaterials. In contrast, younger patients or those with fewer comorbidities may have a more efficient antioxidative response, which could affect the material's responsiveness and drug release kinetics. Given these factors, it is critical to design ROS-responsive biomaterials that are not only effective in a general population but also customizable to individual patients' unique pathological and physiological characteristics. This could involve designing systems that can adapt to varying levels of ROS and inflammatory mediators, considering different patient profiles for more personalized and effective treatment regimens.

(5) **Interdisciplinary collaboration:** With the continuous advancement of interdisciplinary collaboration between biomaterials science, nanotechnology, and medicine, the design and optimization of ROS-responsive biomaterials have entered a new era of development. The rapid progress of artificial intelligence (AI) technologies, combined with big data analytics and machine learning algorithms, has ushered in a new phase for the design and optimization of ROS-responsive biomaterials, opening unprecedented possibilities for the precise treatment of CVDs. AI can be leveraged for the efficient design of ROS-responsive materials, especially in the context of personalized and precise interventions for disease treatment. Through machine learning algorithms, AI can extract optimal design parameters for materials—such as structure, chemical composition, and response mechanisms—from large datasets, accelerating the material development process. For example, AI can analyze the dynamic changes of ROS in different CVDs and, by integrating big data analysis, design materials that respond in specific oxidative environments. The application of AI can effectively optimize the selectivity and response rate of materials, allowing them to better adapt to the pathological microenvironment of cardiovascular lesions and achieve more precise drug release and treatment.

In conclusion, by advancing ROS-responsive biomaterials, researchers pave the way for innovative diagnosis and treatment that could revolutionize CVDs care. Through optimized design and interdisciplinary collaboration, these materials hold great potential to improve diagnosis, treatment efficacy, and patient outcomes, offering new avenues for personalized, effective therapies in CVDs management.

## Figures and Tables

**Table 1:** ROS-responsive biomaterials in CVDs.

Types of Diseases	Types of Biomaterials	Biomaterials	ROS-responsive Moieties	Therapeutic Mechanism	Ref.
Thrombotic Disorders	Nanocoating	PBA Fiber	Arylboronic Ester	Anti-Inflammatory, Reduces Thrombosis Formation	124
	Hydrogel	TEMPO	Arylboronic Ester	Anticalcification, Endothelialization, Anticoagulation	126
	Nanomicelle	BCMs	Arylboronic Ester	ROS Scavenging, Anti-Inflammatory	130
	Nanoparticle	HDP	Ferrocene	Anti-Inflammatory, Anticoagulant	131
	Nanovesicle	IDM&NK@PPTV	Arylboronic Ester	Anti-Inflammatory, Antithrombotic	132
Atherosclerosis	Nanomicelle	PEG-PPS	PPS	Reduces Inflammation and ROS Levels	138
	Nanoparticle	SV MC@RBCs	PPS	Reduces ROS Levels, Antithrombotic	139
	Nanomicelle	PEG-Ptyr-EO	Ferrocene	Inhibits Pro-Inflammatory Macrophage Accumulation, Reduces Oxidative Stress	140
	Nanoparticle	RPP-PU	Ferrocene	Scavenges H_2_O_2_, Lowers Serum LDL	35
	Nanoparticle	OEM@RAP NPs	Ferrocene	Inhibits Vascular Plaque Progression, Suppresses Smooth Muscle Cell Proliferation	141
	Nanoparticle	RBC/LFP@PMMP	Ferrocene	Anti-Inflammatory, Inhibits Foam Cell Formation	142
	Nanoparticle	PF/TC-AT-d-rHDL	Peroxalate Ester	Cholesterol Scavenging, Reduces Cellular Lipid Deposition, Promotes M2 Polarization	145
	Nanoparticle	KPF@MM-NP	Ferrocene	Inhibits Pro-Inflammatory Factor Expression, Promotes M2 Polarization	146
	Nanoparticle	MM@CD-PBA-RVT	3-Nitrophenylboronic Acid	Promotes transition of Macrophages from M1 to M2, Facilitates the Removal of Lipids	147
	Nanoparticle	MacTNP	HA	Macrophage Cytotoxicity	148
	Nanomicelle	TS-IIA-PM	Arylboronic Ester	Inhibits LDL Oxidation, Reduces Oxidative Stress	150
	Nanostructure	HA-Fc/NP^3^_ST_	Peroxalate Ester	Increases Cholesterol Efflux, Anti-Inflammatory	29
Myocardial Infarction	Cardiac Patch	PFTU/GT	Thioketal	Reduces ROS Levels and Lipid Peroxidation	157
	Nanoparticle	TPCD-NPs	Peroxalate Ester	Inhibits Oxidative Stress and Cellular Damage	158
	Hydrogel	Gel-bFGF	Arylboronic Ester	Inhibits Cardiomyocyte Apoptosis, Improves Cardiac Function	161
	Nanoparticle	PEG-PPS-PEG@MR409 NPs	PPS	Reduces Myocardial Cell Apoptosis	162
	Hydrogel	OGDPR	Arylboronic Ester	Scavenges ROS, Inhibits Apoptosis, Suppresses Myocardial Fibrosis	163
	Nanoparticle	HSD-R	Arylboronic Ester	Inhibits Pro-Apoptotic genes, Attenuates Local Inflammation	164
	Hydrogel	HB-PBAE/HA-SH/TIIA@PDA	Disulfide Bond	Inhibits Ventricular Dilation, Reduces Inflammatory Factor Expression	196
	Cardiac Patch	PUTK	Thioketal	Increases Ejection Fraction, Reduces Infarct Size, Enhances Myocardial Revascularization	32
Ischemia-Reperfusion Injury	Hydrogel and Nanoparticle	PTPSC	Arylboronic Ester	Restores Mitochondrial Function, Alleviates Oxidative Stress	171
	Nanoparticle	PEG-b-PPS-Rg3	PPS	Enhances Mitochondrial Autophagy, Antioxidative Stress	172
	Nanoparticle	HPOX	Ferrocene	Antioxidant, Anti-Inflammatory	38
	Nanoparticle	PLP-RvD1	Diselenide	Improves Cardiac Remodeling, Promotes Angiogenesis	26
	Nanoparticle	Lipo^PEP-20^	Diselenide	Inhibits the Initiation and Promotes Active Resolution of Inflammation	177
	Hydrogel	CS-B-NO	Arylboronic Ester	Regulates ROS/NO Balance	178
	Nanoparticle	M/PCOD@PLGA	/	Inhibits Inflammation and Reduces Cell Apoptosis	176
Vascular Restenosis	Nanoparticle	Ox-bCD	Arylboronic Ester	Inhibits VSMCs Migration and Proliferation	183
	Nanoparticle	RAP/AOCD	Arylboronic Ester	Inhibits VSMCs Migration and Proliferation	184
	Nanoparticle	MM@PCM/RAP	Arylboronic Ester	Inhibits VSMCs Proliferation	185
	Nanoballoon	PBC	Arylboronic Ester	Antioxidant, Anti-inflammatory, Endothelial Protection	186
	Nanocluster	ROS-Detonable Nanocluster	Arylboronic Ester	Inhibits VSMCs Migration and Proliferation	187
Peripheral Artery Disease	Hydrogel	PHB-DEX	Arylboronic Ester	Downregulates Inflammatory Gene Expression	41
	Nanoparticle	3S-PLGA-po-PEG	Ferrocene	Promotes Angiogenesis, Anti-Inflammatory, Cardiovascular Protection	190
Vascular Endothelial Cell Dysfunction	Nanoparticle	RBC-LVTNPs	Peroxalate Ester	Improves Endothelial Cell Function, Inhibits Inflammatory Factors	193
Abdominal Aortic Aneurysms	Nanoparticle	CROR NPs	4-(Hydroxymethyl)phenylboronic Acid Pinacol Ester	Inhibits Calcification, Attenuates ROS-Mediated Oxidative Stress and Apoptosis	194
	Nano-Micelle	TPTN	4-Hydroxyphenylboronic Acid Pinacol Ester	Inhibits Migration and Activation of Inflammatory Cells	195

## Abbreviations

AAA: Abdominal aortic aneurysm
AI: Artificial intelligence
AIE: Aggregation-induced emission
AIEgens: Aggregation-induced emission luminophores
Andro: Andrographolide
AS: Atherosclerosis
ASO: Antisense oligonucleotide
BCMs: PEGylated poly(carbonate)s micelles
bFGF: Basic fibroblast growth factor
BPNSs: Black phosphorus nanosheets
BTZ: Bortezomib
CA: Caffeic acid
CDs: Carbon fluorescent quantum dots
CO_2_: Carbon dioxide
CsA: Cyclosporin A
CVDs: Cardiovascular diseases
CYP2J2: Cytochrome P450 epoxygenase 2J2
DDS: Drug delivery system
DES: Drug-eluting stents
DHA: Docosahexaenoic acid
d-rHDL: Discoidal reconstituted high-density lipoprotein
ECM: Extracellular matrix
ECs: Endothelial cells
FC: Fe_3_O_4_@SiO_2_-CDs
Fc: Ferrocene
H_2_O_2_: Hydrogen peroxide
H_2_S: Hydrogen sulfide
HA: Docosahexaenoic acid
HA: Hyaluronic acid
HA-SH: Thiol-modified hyaluronic acid
HBA: Hydroxybenzyl alcohol
p-HBA: Para-hydroxybenzyl alcohol
HPOX: Polyoxalate copolymer
ICG: Indocyanine green
IKK: I-κB kinase
IO@CO: Iron oxide/cerium oxide
IONPs: Iron oxide nanoparticles
iPC: Intrapericardial
I/R: injury Ischemia-reperfusion injury
IS: Ischemic stroke
KPF: Kaempferol
LDL: Low-density lipoprotein
LFP: Lipid-specific probe
LV: Left ventricle
LVEDV: Left ventricular end-diastolic volume
LVEF: Left ventricular ejection fraction
LVESV: Left ventricular end-systolic volume
LVT: Lovastatin
MI: Myocardial infarction
MIRI: Myocardial ischemia-reperfusion injury
MM: Macrophage membranes
MMP: Matrix metalloproteinases
MNPs: Magnetic nanoparticles
MP: Methylprednisolone
mPTP: Mitochondrial permeability transition pores
MRI: Magnetic resonance imaging
MSNs: Mesoporous silica nanocarriers
NF-κB: Nuclear factor-κB
NIR-II: Second near-infrared
NO: Nitric oxide
NPs: Nanoparticles
Olfr2: Olfactory receptor 2
ox-Dex: Oxidized dextran
ox-LDL: Oxidized LDL
PAD: Peripheral artery disease
PAI: Photoacoustic imaging
PBA: 3-nitrophenylboronic acid
PBA: Poly(2-(4-((2,6-dimethoxy-4-methylphenoxy) methyl) phenyl)-4,4,5,5-tetramethyl-1,3,2-dioxab)
PBAP: phenylboronic acid pinacol ester
PBMEO: Bis(methoxyethoxymethyl) ether oxide
PCI: Percutaneous coronary intervention
PDA: Polydopamine
PDGF: Platelet-derived growth factor
pDNA: Plasmid DNA
PDT: Photodynamic therapy
PEG-b-PPS: Poly (ethylene glycol)-b-Poly (propylene sulfide)
PEG-Ptyr-EO: Poly (ethylene glycol)-poly (tyrosine-ethyl oxalate)
PGMA-PEG: Poly (glycidyl methacrylate)-polyethylene glycol
phS: Phenylene sulfide groups
PLM: Platelet cell membrane
PM: Platelet membrane
PMM: Poly (2-methacryloyloxyethyl phosphorylcholine
PPIS@FC: PGMA-PEG-ISO-1-Sim@FC
Pred: Prednisone
PUTK: Polyurethane
PVA: Polyvinyl alcohol
RA: Rosmarinic acid
RAP: Rapamycin
RBCM: Red blood cell membrane
RBCs: Red blood cells
rHDL: Reconstituted high-density lipoprotein
ROS: Reactive oxygen species
RvD1: Resolvin D1
SAA: Salvianolic acid A
SAB: Salvianolic acid B
Sim/SV/SIM/STx: Simvastatin
si-Olfr2/Anti-Olfr2: siRNA
SOD: Superoxide dismutase
SPM: Specialized pro-resolving mediator
T IIA/ TS-IIA: Tanshinone IIA
TF: Tissue factor
TMSN: Tempol-grafted MSN
TNF-α: Tumor necrosis factor-alpha
TPCDP: Two-photon fluorophore cyclodextrin/prednisone complex
TXA2: Thromboxane A2
VA: Vanillyl alcohol
VEGF: Vascular endothelial growth factor
VSMC: Vascular smooth muscle cells
β-CD: β-cyclodextrin
·OH: Hydroxyl radical
